# Trans-activator of transcription-pre-B-cell leukemia transcription factor 1 alleviates Alzheimer’s disease by reducing neuronal insulin resistance and restoring energy homeostasis

**DOI:** 10.4103/NRR.NRR-D-25-01313

**Published:** 2026-02-05

**Authors:** Xiangyuan Meng, Zinan Liu, Zhenhu Zhao, Lei Chen, Siyao Li, Qi Song, Ruihan Guo, Xin Zhang, Fanlei Meng, Hui Zhang, Li Tan, Xinpeng Liu, Yujie Wang, Feng Zhong, Run Liu, Tianlin Gao, Jinyu Liu

**Affiliations:** 1 School of Public Health, Jilin University, Changchun, Jilin Province, China; 2 School of Health and Life Sciences, University of Health and Rehabilitation Sciences, Qingdao, Shandong Province, China; 3 Institute of Agricultural Quality Standard and Testing Technology, Jilin Academy of Agricultural Sciences, Changchun, Jilin Province, China; 4 School of Public Health, Qingdao University, Qingdao, Shandong Province, China

**Keywords:** Alzheimer’s disease, amyloid-beta oligomers, amyloid precursor protein/presenilin 1, insulin receptor substrate 1, insulin resistance, metabolic reprogramming, molecular medicine, neuron, oxidative phosphorylation, pre-B-cell leukemia transcription factor 1

## Abstract

Alzheimer’s disease is characterized by hippocampal neuronal apoptosis, which leads to cognitive decline. The pathophysiology of Alzheimer’s disease is largely driven by disrupted cellular metabolism and insufficient neuronal energy supply. However, current treatments for Alzheimer’s disease remain limited because of side effects and disease complexity. Increasing evidence suggests that amyloid-β oligomer-induced neuronal insulin resistance and metabolic dysfunction play key roles in Alzheimer’s disease progression, yet their underlying mechanisms and therapeutic strategies remain unclear. In this translational preclinical study, we combined a case-control analysis, *in vitro* cell assays, and *in vivo* experiments using amyloid precursor protein/presenilin 1 transgenic mice to investigate whether transcriptional regulation of key regulatory factors restores neuronal energy supply and improves Alzheimer’s disease-induced pathology. The case-control analysis identified pre-B-cell leukemia transcription factor 1 as a crucial regulator of brain metabolic homeostasis in Alzheimer’s disease. We developed a blood–brain barrier-permeable trans-activator of transcription-pre-B-cell leukemia transcription factor 1 fusion protein to enhance pre-B-cell leukemia transcription factor 1 expression, with the aim of restoring neuronal energy supply and reducing apoptosis. Mechanistic investigations using Alzheimer’s disease models revealed that pre-B-cell leukemia transcription factor 1 transcriptionally upregulates insulin receptor substrate 1 by interacting with its promoter, which resulted in augmented insulin signaling. Trans-activator of transcription-pre-B-cell leukemia transcription factor 1 downregulated PDK4, significantly upregulated the expression of pyruvate dehydrogenase, and promoted mitochondrial oxidative phosphorylation activity, which inhibited the abnormally enhanced glycolytic flux, increased adenosine triphosphate production, and ultimately helped restore neuronal energy homeostasis. Therapeutic administration of trans-activator of transcription-pre-B-cell leukemia transcription factor 1 in amyloid precursor protein/presenilin 1 transgenic mice significantly enhanced cognitive performance, diminished hippocampal neuronal apoptosis, and mitigated amyloid-β deposition. No significant hepatotoxicity, nephrotoxicity, or other detectable adverse effects were observed within the dose ranges and time windows of administration. Taken together, we identified pre-B-cell leukemia transcription factor 1 as a crucial transcriptional regulator of neuronal energy metabolism in Alzheimer’s disease. Moreover, we elucidated the molecular mechanism through which the pre-B-cell leukemia transcription factor 1–insulin receptor substrate 1 signaling axis sustains metabolic homeostasis. Furthermore, we demonstrated that the blood–brain barrier-permeable trans-activator of transcription-pre-B-cell leukemia transcription factor 1 fusion protein constitutes a mechanistically innovative and highly translatable therapeutic strategy for Alzheimer’s disease.

## Graphical abstract

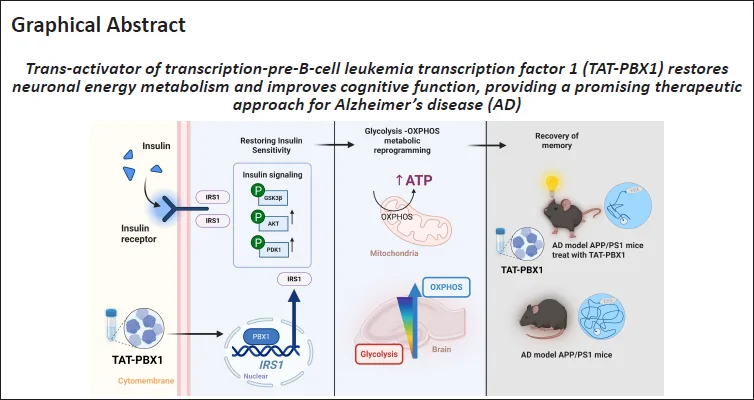

## Introduction

In recent years, novel technologies and theoretical frameworks have focused on neuronal metabolic dysfunction; moreover, the reestablishment of energy homeostasis has emerged as a central theme in neurodegenerative disease research (Anselme et al., 2025; Meng et al., 2025). In this field, research into neuronal energy crises and the factors that regulate them has become a major focus for the development of new treatment methods. Furthermore, such crises are considered important starting points for understanding the mechanisms underlying neurodegenerative diseases, such as Alzheimer’s disease (AD). However, owing to the side effects of drugs and the natural complexity of the disease, current treatments for AD remain largely ineffective (Rabinovici, 2021). Given the accelerating global trend of population aging, gaining a better understanding of AD pathogenesis and identifying effective therapeutic targets have become critical priorities in public health research (Hayat et al., 2025).


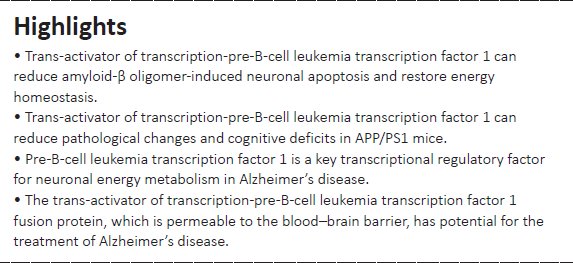


Although extensive research has recognized metabolic dysfunction—especially the neuronal energy crisis—as an early and significant pathological occurrence in AD, debate regarding its fundamental causes, essential regulatory points, and potential reversibility persists, which highlights the urgent need for more comprehensive investigations (Zhang et al., 2024). Neurons are the primary loci for memory formation, and neuronal damage is the cause of the primary symptoms of AD. Neurons predominantly depend on oxidative metabolism, whereby glucose is needed to produce adenosine triphosphate (ATP) via oxidative phosphorylation (OXPHOS) for effective synaptic transmission and functional maintenance (Wolfe et al., 2024; Swain et al., 2025). During the onset and progression of AD, neurons experience an aberrant and increasingly decompensated phase of metabolic reprogramming, which is primarily marked by an early and escalating dysfunction of mitochondrial OXPHOS (Minhas et al., 2024). This compels neurons to compensate for their dependence on glycolysis to maintain their energy supply. Ultimately, the ongoing energy crisis triggers intrinsic apoptotic signaling pathways, which results in neuronal apoptosis and cognitive decline (Ozgen et al., 2022). Therefore, targeting neuronal glycolytic flux may aid in restoring neuronal energy homeostasis, augmenting ATP production to facilitate neuronal function, and diminishing neuronal apoptosis, thereby presenting a potential therapeutic strategy for AD.

In the central nervous system, insulin not only regulates blood glucose but also serves as a critical signal for maintaining neuronal survival, synaptic plasticity, and energy metabolism (Zhu et al., 2022; Hees et al., 2024). When insulin resistance develops in the brain (characterized by reduced neuronal sensitivity to insulin), glucose uptake and utilization are severely impaired, which consequently drives neurons toward an energy crisis (Milstein and Ferris, 2021). Insulin receptor substrate 1 (IRS1) is a key node in the insulin signaling pathway and is closely associated with the development of insulin resistance (Toyoshima et al., 2025). However, under AD pathological conditions, IRS1 undergoes severe dysfunction, with abnormally elevated phosphorylation at S616 (S612 in mice) (Ma et al., 2009; Talbot et al., 2012) This aberrant modification directly disrupts normal insulin signaling, which leads to transcriptional repression via the release of the downstream metabolic enzyme pyruvate dehydrogenase kinase 4 (PDK4) (Connaughton et al., 2010). In turn, there is a marked reduction in neuronal insulin sensitivity, which impedes glucose oxidation into the tricarboxylic acid (TCA) cycle, ultimately weakening mitochondrial OXPHOS capacity and causing neuronal energy failure. PDK4 is a central regulator of cellular OXPHOS and glycolysis and is highly expressed in patients with diabetes and obesity (McAinch et al., 2015; Dekkar et al., 2025). Although several recent studies have shown that pharmacological inhibition of PDKs or insulin treatment can partially reduce neuronal apoptosis (Craft et al., 2020; Ghareghani et al., 2022; Huang et al., 2025), the potential peripheral neurotoxicity associated with long-term use of PDK inhibitors, as well as decreased insulin sensitivity induced by insulin therapy, must be carefully considered (Wu et al., 2025). Therefore, upregulating IRS1 expression may represent an effective treatment strategy for AD for restoring neuronal insulin resistance, reducing glycolysis, promoting neuronal OXPHOS, reestablishing energy homeostasis, and supporting neuronal survival.

Pre-B-cell leukemia transcription factor 1 (PBX1) is a member of the three-amino acid loop extension homeobox transcription factor family and a key regulator of adult neurogenesis in the subventricular zone. Additionally, PBX1 plays an active role in neural progenitor cell fate determination and cellular reprogramming (Jiang et al., 2019; Hau et al., 2021). The overexpression of PBX1 mitigates apoptosis induced by 6-hydroxydopamine in SH-SY5Y cells (Li et al., 2022). Genome-wide association studies of both European and Asian populations have identified PBX1 as a biological candidate gene for metabolic syndrome and type 2 diabetes mellitus (T2DM), underscoring its regulatory role in insulin homeostasis (Ban et al., 2008; Duesing et al., 2008). In our previous work, we showed that PBX1, along with the downstream pluripotency factor NANOG, activates the protein kinase B (AKT)/glycogen synthase kinase 3 (GSK3) β signaling pathway, which sustains energy supplies during proliferation and promotes cell growth (Jiang et al., 2019). Although it is well established that PBX1 plays a regulatory role in neurogenesis and metabolic control, it remains unclear whether it directly modulates insulin sensitivity and neuronal energy homeostasis.

In this study, we aimed to examine whether PBX1 modulates neuronal energy homeostasis by reinstating neuronal insulin sensitivity and, in turn, promotes neuronal survival. To produce non-modified exogenous proteins, prokaryotic expression systems are ideal because they are economical, effective, easy to use, and have well-established methods (Schütz et al., 2023). We successfully constructed a trans-activator of transcription (TAT)-PBX1 fusion protein using a prokaryotic expression system. The fusion protein retained the transcriptional activity of PBX1 while enabling efficient cellular uptake. *In vitro* and *in vivo* AD models were established using amyloid-β oligomers (AβOs) and APP/PS1 mice, respectively. We demonstrated that PBX1 directly binds to and activates IRS1, increasing its transcriptional activity and thereby restoring neuronal insulin sensitivity in the context of an AβO-induced impairment. The therapeutic potential of TAT-PBX1 was evaluated in APP/PS1 mice, with a focus on its effects on cognitive function, brain insulin resistance, and cerebral energy metabolism. Our aim was to clarify this novel mechanism to contribute to the development of therapeutic strategies and offer guidance in the prevention and treatment of AD.

## Methods

### Case-control study

To preliminarily investigate the relationship between PBX1 and human cognitive function, we conducted a case-control study in Licha Town, Jiaozhou, Qingdao, Shandong Province. All participants provided informed consent, and the study protocol was approved with a commitment to strict confidentiality. All data collected were used solely for scientific research purposes and were not disclosed publicly. All procedures performed in the study were conducted in accordance with the ethical standards outlined in the 1964 *Declaration of Helsinki*, as revised in 2013. The human study protocol complied with the ethical principles outlined in the *Declaration of Helsinki*, was registered in the Chinese Clinical Trial Registry (No. ChiCTR2300078236), and approved by the Ethics Committee of the School of Public Health, Jilin University, China (approval No. 20240716) on July 22, 2024.

#### Participants

Individuals aged over 60 years who were conscious, able to communicate normally, had resided locally for more than 6 months, and met the diagnostic consensus criteria for mild cognitive impairment (MCI; encompassing subjective cognitive decline and objective cognitive test impairment with largely preserved activities of daily living but not meeting the diagnostic criteria for dementia) were included in the study. Controls (CON) were selected from the same villages or towns, had no history of neurological or psychiatric disorders, and scored above the education-adjusted cutoff thresholds for the Mini-Mental State Examination (MMSE). Individuals with previously diagnosed diabetes, major depressive disorder, other psychiatric conditions, severe hepatic or renal diseases, recent alcohol abuse, hearing impairment, communication difficulties, or inability to complete questionnaires were excluded. The final analysis comprised 73 patients diagnosed with MCI and 127 age-matched cognitively normal CONs, which resulted in a case-control ratio of 1:1.7. The study subjects constituted all eligible participants who were identified and available for enrollment during the predefined recruitment period.

#### Data collection and clinical laboratory assessments

T2DM was diagnosed according to the criteria of the International Diabetes Federation: a fasting blood glucose (FBG) concentration of ≥ 7.0 mM, a 2-hour plasma glucose concentration during an oral glucose tolerance test of ≥ 11.1 mM, and/or a glycated hemoglobin (HbA1c) concentration of ≥ 6.5%. Individuals meeting any of these thresholds were excluded from the study to ensure none of our patients had T2DM (American Diabetes Association, 2021). MCI was diagnosed according to neuropsychological assessment via expert consensus (Neurology, 2025). Briefly, trained senior medical students or healthcare staff at local health centers conducted face-to-face interviews using the MMSE. The data were reviewed by neurologists specializing in cognitive disorders. All interviewers and healthcare workers underwent standardized training over 1 week that encompassed demographic data collection, lifestyle evaluation, and medical history documentation. The MMSE is a 30-point instrument widely used in clinical and research settings for cognitive screening to assess multiple cognitive domains, such as orientation, immediate recall, attention and calculation, delayed recall, language, and praxis (Arevalo-Rodriguez et al., 2021). We applied the following education-specific cutoff scores: ≤ 17 for illiterate individuals, ≤ 20 for those with primary school education, and ≤ 24 for those with junior high school education or higher. Fasting blood samples were collected after a minimum 10-hour overnight fast. The supernatant obtained after blood centrifugation was used for the biochemical analysis. The biochemical parameters analyzed were: HbA1c, FBG, serum triglyceride (TG), total cholesterol (TC), high-density lipoprotein cholesterol (HDL-C), low-density lipoprotein cholesterol (LDL-C), alanine aminotransferase (ALT), aspartate aminotransferase (AST), lactate dehydrogenase (LDH), creatine kinase (CK), creatine kinase-myocardial band (CK-MB), total bilirubin, serum creatinine. All assays were performed using a Beckman AU5800 automatic biochemical analyzer (Beckman Coulter, Miami, FL, USA).

The levels of metabolites in human serum were quantified using liquid chromatography-tandem mass spectrometry, as previously described (Chen et al., 2025). Briefly, samples were extracted with precooled lysis buffer (acetonitrile/methanol/water ratio = 2:2:1, precooled to –20°C), vortexed thoroughly, and centrifuged at 17,000 × *g* for 10 minutes at 4°C. A total of 150 μL of supernatant was transferred for analysis. Chromatographic separation was performed using a ZORBAX SB-C18 column (2.1 mm × 100 mm, 5 μm; Agilent, Santa Clara, CA, USA) at 35°C. Mass spectrometry was conducted in negative ion mode, and data were acquired in centroid mode.

#### Bias

Several steps were taken to minimize bias. Interviewers underwent the same training procedure, and all collected data were checked daily. In addition, all data were entered using a double-data entry method. There was no missing data for any of the variables of interest; thus, all recruited subjects were included in the final analysis. This reduced the risk of selection and information bias.

#### Cell culture and treatment

The human neuroblastoma SH-SY5Y cell line was purchased from the Chinese Academy of Sciences Cell Bank (SCSP-5014, Shanghai, China) and cultured in a 1:1 mix of Minimum Essential Medium (MEM; 11095080, Gibco, Grand Island, NY, USA)/F12 medium (11765054, Gibco), supplemented with 10% (v/v) fetal bovine serum (12103C; Sigma, St. Louis, MO, USA), 100 U/mL penicillin-streptomycin (SC118-01; Seven, Beijing, China), and 1% nonessential amino acids (M7145; Sigma). All cells were maintained in a humidified incubator at 37°C with 5% carbon dioxide. For treatment, AβOs and/or TAT-PBX1 prepared under different incubation conditions were diluted in MEM/F12 and cultured with the cells for 24 hours.

#### Establishment of an insulin resistance model

To induce insulin resistance, SH-SY5Y cells were cultured in a serum-free 1:1 mix of MEM (11095080, Gibco)/F12 medium (11765054, Gibco), supplemented with 1 μM insulin (PB180432, Procell, Wuhan, China) for 24 hours. Following incubation, the medium was removed, and the cells were washed three times with phosphate buffer solution (PBS). Cells were then incubated for 30 minutes in a serum- and insulin-free MEM/F12 medium, followed by stimulation with insulin at a final concentration of 10 nM for 15 minutes. Cells were harvested for insulin sensitivity analysis. A failure to observe increased ratios of phosphorylated Akt at Ser473 (p-AKT S473) to total AKT and phosphorylated glycogen synthase kinase 3 beta (GSK3β) at Ser9 (p-GSK3β S9) to total GSK3β following acute insulin stimulation (10 nM for 15 minutes) was considered to indicate insulin resistance in SH-SY5Y cells.

### Animal experiments

#### Animals

Twenty-four male APPswe/PSEN1dE9 (hereafter referred to as APP/PS1) transgenic mice (age, 10 months; body weight, 26–28 g), 12 male C57BL6J mice (age, 10 months; body weight, 26–28 g), and 54 male C57BL/6J mice (age: 8 weeks; body weight: 20–23 g) were purchased from Beijing Huafukang Biotechnology Co., Ltd. (Beijing, China; license number: SCXK [Jing] 2024-0003). The animal experiment was conducted at the facilities of the School of Public Health, Jilin University (SYXK [Ji] 2021-0003) under the supervision of the Institutional Animal Care and Use Committee of Jilin University. All procedures complied with the GB2018-35892 Guidelines for Ethical Welfare Review of Laboratory Animals, and housing conditions adhered strictly to GB14925 standards. All experiments were designed and reported according to the National Institutes of Health Guide for the Care and Use of Laboratory Animals (8^th^ edition; National Research Council, 2011). All animal procedures were approved and supervised by the Animal Ethics Committee of the School of Public Health, Jilin University (approval No. KT202407005), on July 18, 2024, and followed established ethical policies and protocols.

All mice were housed in a climate-controlled environment (22.8 ± 2.0°C, 45%–50% humidity) on a 12-hour light/dark cycle and provided with ad libitum access to tap water and standard laboratory chow. All procedures conformed to the National Institutes of Health Guide for the Care and Use of Laboratory Animals. Only male mice were used in this study to avoid sex-related differences in AD-related cognitive impairment (Vest and Pike, 2013). A previous study has shown that APP/PS1 transgenic mice begin to exhibit significant deficits in learning and memory at 10 months of age (Yang et al., 2022). Therefore, 10-month-old male APP/PS1 mice and age-matched nontransgenic wild-type (WT) littermates were specifically selected for the study.

#### Selection of pharmacological treatment regimen

According to a previous study protocol, it was determined whether TAT-PBX1 can cross the blood–brain barrier and reach the brain by first conducting pharmacokinetic analyses (Glasenapp et al., 2025). Eight-week-old male C57BL/6J mice were subjected to adaptive feeding for 1 week. After adaptive feeding, the mice were randomly divided into an intraperitoneal injection (i.p.) group or an oral gavage (i.g.) group according to average body weight. Mice in the two groups were then administered TAT-PBX1 via i.p. injection (1.5 mg/kg) or i.g. (1.5 mg/kg). Blood and brain samples were collected at 0, 5, 15, and 30 minutes and 1, 2, 4, 8, and 24 hours after administration (**Additional Figure 1**). Following either i.p. or i.g. administration, the concentration of TAT-PBX1 peaked in brain tissue at 4 hours, whereas that in plasma peaked at 1 hour (**Additional Figure 1** and **Additional Table 1**). The mean residence time, which was calculated from the area under the curve (0–t) and area under the moment curve, indicated longer brain exposure with i.p. administration (26.59 hours in the brain and 2.42 hours in plasma) than with i.g. administration (19.64 hours in the brain and 1.96 hours in plasma; **Additional Table 1**). Because a suitable reference formulation was unavailable, we did not assess relative bioavailability. Our results confirmed that TAT-PBX1 effectively reaches the brain in rodents and that i.p. administration results in superior delivery efficiency over i.g. administration. Therefore, i.p. injection was chosen as the route of administration for subsequent treatments.

**Additional Table 1:** Pharmacokinetic parameters of TAT-Pre-B-cell leukemia transcription factor 1 (PBX1) in mice following intraperitoneal (i.p.) injection and oral gavage administration

Matrix		Brain		Plasma	

Administration Route		i.p.	i.g.	i.p.	i.g.

Parameters	Unit				
Dose level	mg/kg	1.5	1.5	1.5	1.5
T_1/2_	h	10	8	2	2
T_max_	h	4	4	1	1
C_max_	ng/g, ng/mL	11.65	8.05	22.31	11.09
AUC_(0-t)_	h*ng/g, h*ng/mL	73.85	40.85	117.6	55.63
AUC_(0-∞)_	h*ng/g, h*ng/mL	107.76	51.48	147.45	60.65
AUMC	ng-h^2^/mL	1963.72	802.47	285.16	108.76
MRT	h	26.59	19.64	2.42	1.96
λz	/h	0.069	0.087	0.35	0.35
CL	ng/h/g, mL/h/Kg	0.014	0.029	0.010	0.025
V_d_	ng/g, mL/Kg	0.20	0.34	0.030	0.071

AUC_(0-∞)_: The area under the concentration-time curve extrapolated to infinity; AUC_(0-t)_: The area under the curve from time zero to the last measurable concentration; AUMC: area under the moment curve; CL: clearance; Cmax: the maximum concentration; i. g.: intragastric; MRT: mean residence time; T1/2: the elimination half-life; Tmax: time to reach maximum plasma concentration; Vd: volume of distribution. λz: the terminal elimination rate constant.

#### Groups

The WT mice were randomly assigned to the control (WT; *n* = 6; no treatment) or vehicle control group (PBS; *n* = 6; WT mice i.p. injected with 0.2 mL of PBS) according to average body weight. APP/PS1 mice were randomly divided into four groups: the model group (APP/PS1, *n* = 6; no treatment), low-dose TAT-PBX1 group (APP/PS1 + TP low, 1.0 mg/kg TAT-PBX1), medium-dose TAT-PBX1 group (APP/PS1 + TP mid, 1.5 mg/kg), and high-dose TAT-PBX1 group (APP/PS1 + TP hi, 2 mg/kg) according to average body weight. Injections were administered every other day for a total of 10 doses. Humane endpoints for the experimental animals included, but were not limited to, the following: animals nearing death or unable to move; no response to gentle stimulation; a body weight loss of > 20% from the pre-experiment weight; inability to eat or drink; and signs of significant anxiety or restlessness. At the end of the study, the mice were anesthetized; blood samples were collected; and the brains, livers, and kidneys were harvested.

#### Behavioral experiments

All behavioral tests were conducted in ascending order of stress level, from lowest to highest, to minimize interference from preceding tests on subsequent results. The testing sequence was as follows: Open Field Test (OFT), followed by the Y-maze test, and finally the Morris Water Maze (MWM) test.

*Open Field Test*

The OFT was performed after the 10^th^ administration of TAT-PBX1 and was used to evaluate spontaneous exploratory behavior in mice using previously established protocols (Seibenhener and Wooten, 2015). Briefly, the mice were acclimated to the behavioral testing room for 3 days before the experiment. One hour before testing, the room lights were turned off, and the room was kept quiet. Each mouse was gently placed into a corner of the open field arena (25 cm × 25 cm × 40 cm), and an automated tracking system (SANS, Jiangsu, China) was activated. The mice were observed for 5 minutes, during which the distance traveled and time spent in the central area were recorded. The ratio of central area activity was calculated as (time in center/total time) × 100%. After each test, the mice were returned to their home cages, and the arena was cleaned with 75% ethanol to eliminate olfactory cues.

*Y-Maze test*

The mice were tested in the Y-maze test 72 hours after the OFT. The Y-maze test was performed to evaluate spatial working memory and spontaneous alternation behavior, following established protocols (Kim et al., 2023). The Y-maze test comprised two parts: the spontaneous alternation task and the new arm task. For the spontaneous alternation task, the mice were placed in the middle of the Y-maze and given 8 minutes to explore all three arms on their own. We recorded the order in which the arms were entered and used the formula, [(number of alternations)/(total arm entries – 2)] × 100% to find the percentage of spontaneous alternations. During the training phase of the novel arm task, a partition blocked access to the novel arm to allow entry into only the two familiar arms. The mice were placed in the middle of the maze and given 10 minutes to explore. After that, their behavior was recorded. The partition was then removed during the testing phase so that all three arms could be entered. We recorded the number of entries and time spent in each arm. The exploration ratio for the new arm was calculated using the formula (time in new arm/total exploration time) × 100%. After the tests, the mice were placed back into their cages, and the maze was cleaned with 75% ethanol to eliminate any olfactory cues.

*Morris Water Maze test*

Upon completion of all the dry maze tests, animals were allowed a 5-day rest period before proceeding to the MWM test. The MWM test is used to assess learning and spatial memory, and the experimenters were blinded to the treatment conditions of the mice (Chen et al., 2000). The circular pool was filled with water mixed with titanium dioxide powder to render it opaque, and the water temperature was maintained between 22°C and 25°C. A camera connected to a computer was fixed above the center of the pool to record the trajectories of the mice for accurate measurement. The pool was divided into four equal quadrants. The experiment consisted of three phases: the visible platform test, the hidden platform navigation test, and the spatial probe test.

(1) Visible platform test: On day 1, the platform was placed 0.5 cm above the water surface in a randomly selected quadrant. The mice were gently placed into the water at the pool edge, facing the wall. The time to locate the visible platform and the swimming speed were recorded. After each trial, the mice were placed in a warm, clean cage for at least 30 minutes before the next test.

(2) Hidden platform navigation test: From days 2 to 7, the platform was submerged 0.5 cm below the water surface. The mice were tested four times per day, beginning from a different quadrant in each test. The procedure was similar to that of the visible platform test, and the latency to locate the hidden platform and swimming speed were recorded.

(3) Spatial probe test: On day 8, the platform was removed. The mice were allowed to swim freely for 60 seconds. The number of times the original platform location was crossed, the time spent in the target quadrant, and the latency to the first platform crossing were recorded.

### Preparation of amyloid-β1–42 oligomers

Amyloid-β1–42 (Aβ_1–42_, hereafter collectively referred to as Aβ) oligomers were prepared according to a previously established protocol (Stepanov et al., 2022; Chen and Guo, 2023). Briefly, Aβ-lyophilized powder (purity > 95%, high-performance liquid chromatography grade, P9001, Beyotime, Shanghai, China) and pre-chilled hexafluoroisopropanol (HFIP, 105228, Sigma) were kept on ice. HFIP was added to the Aβ to a final concentration of 1 mM and vortexed for 2 minutes, followed by gentle agitation overnight at 4°C to yield a clear Aβ-HFIP solution. Aliquots were transferred to sterile microcentrifuge tubes in a biosafety cabinet and pre-frozen at –80°C for 2 hours. HFIP was then removed by lyophilization for 24 hours (cold trap: –95°C; shelf: –20°C; vacuum: 1 Pa), resulting in a white Aβ-monomer powder, which was stored at –20°C. For aggregation into different Aβ assemblies, the monomer powder was reconstituted in dimethyl sulfoxide (DMSO; D8418, Sigma) at a concentration of 500 μM, vortexed for 10 minutes, and diluted with pre-chilled sterile PBS. The solution was incubated under specific temperature conditions to induce oligomerization. The Aβ monomers were then reconstituted in DMSO before use and incubated under different conditions (i.e., 4°C for 24 hours, 4°C for 48 hours, 37°C for 24 hours, and 37°C for 48 hours) to form AβOs with varying degrees of aggregation.

### Construction of a prokaryotic TAT-PBX1 expression system and recombinant protein denaturation, purification, and refolding

Following a previously reported protocol (Wang et al., 2020), the TAT-PBX1 fusion protein was prokaryotically expressed and purified. The PBX1 coding sequence was polymerase chain reaction (PCR)-amplified from the pLVX-PBX1-IRES-mCherry plasmid, with primers engineered to contain 5’ KpnI and EcoRI sites, digested, and ligated into the pTAT-HA vector. The resultant pTAT-HA-PBX1 plasmid or the empty vector control was transformed into *E. coli* Rosetta (DE3). Transformants were selected on antibiotic-supplemented Luria-Bertani agar, and cultures were expanded in liquid medium. Cells were harvested, lysed via high-pressure homogenization, and the polyhistidine (His)_6_-tagged TAT-PBX1 was purified from the supernatant via nickel-nitrilotriacetic acid chromatography. The eluted protein was refolded by overnight dialysis, followed by buffer exchange and concentration using ultrafiltration. The purity was assessed by sodium dodecyl sulfate -polyacrylamide gel electrophoresis (SDS-PAGE), and the protein identity was confirmed by the detection of the His6 tag, hemagglutinin (HA) tag, and PBX1 epitope via immunofluorescence.

### Measurement of OXPHOS and glycolysis rates

The oxygen consumption (OCR) and extracellular acidification rates (ECAR) were measured using a Seahorse XF24 Extracellular Flux Analyzer (Agilent, Santa Clara, CA, USA), as previously described (Lejri et al., 2025). The assays were performed with the Seahorse XF Cell Mito Stress Test Kit (103015-100, Agilent) and Seahorse XF Glycolysis Stress Test Kit (103020-100, Agilent), following the manufacturer’s protocols. SH-SY5Y cells were seeded into Seahorse XF24 microplates at a density of 1 × 10^4^ cells/well. Before the assay, cells were incubated in Seahorse assay medium at 37°C in a carbon dioxide-free incubator for 1 hour. For mitochondrial stress testing, the sequential injection of compounds was as follows: 1.5 μM oligomycin, 1.5 μM carbonyl cyanide-4 (trifluoromethoxy) phenylhydrazone, and 1 μM rotenone/antimycin A. For glycolysis stress testing, the injections comprised 0.5 μM rotenone/antimycin A, followed by 50 mM 2-deoxy-D-glucose. Data acquisition and analysis were performed using the Wave software (v2.6.4.24, Agilent). Key mitochondrial and glycolytic function parameters were automatically calculated. At the end of the assay, cells from each well were harvested and counted to normalize the OCR and ECAR values to cell number.

### Western blot analysis

Western blot analysis was performed as previously described (He et al., 2022). Briefly, for cell samples, the culture medium was removed, and cells were washed three times with PBS containing a 1% protease inhibitor (P1005, Beyotime), followed by centrifugation. Cells were lysed on ice for 20 minutes in radioimmunoprecipitation assay (RIPA) buffer (SW104, Sevenbio, Beijing, China), supplemented with phenylmethylsulfonyl fluoride (P7626, Sigma), protease inhibitors, and phosphatase inhibitors (SW107, Sevenbio). For mouse brain tissue, samples were ground thoroughly in liquid nitrogen, then lysed in the same ice-cold RIPA buffer for 20 minutes. After vortexing and centrifugation (14,000 × *g* for 20 minutes at 4°C), the supernatant was collected for further analysis. Protein concentrations were determined using a bicinchoninic acid (BCA) protein assay kit (ZJ101, Epizyme, Shanghai, China). Equal amounts of protein were separated by 8%–12% SDS-PAGE and transferred onto polyvinylidene fluoride membranes (IPVH00010, Millipore, Billerica, MA, USA). Membranes were blocked with 5% non-fat dry milk or 5% bovine serum albumin at room temperature for 1 hour to prevent non-specific binding. Subsequently, membranes were incubated overnight at 4°C with primary antibodies specific to the target proteins (**Additional Table 2**). The following day, membranes were washed three times with Tris-buffered saline with Tween-20 and incubated with horseradish peroxidase-conjugated secondary antibodies at room temperature for 2 hours. Protein bands were visualized using an enhanced chemiluminescence substrate. Band intensities were analyzed semi-quantitatively using the ImageJ (version 1.8.0.172, National Institutes of Health, Bethesda, MD, USA) software.

**Additional Table 2:** Primary antibodies used in this study

Antibodies	Manufacturer	Catalog number
6*His tag	Proteintech	66005-1-IG
6E10	Invitrogen	MA5-48043
A11	Invitrogen	AHB0052
Akt	CST	9272
p Akt S473	CST	9271
Complex V	Invitrogen	43-9800
Bax	Selleck	F0037
Bcl-2	Abcam	ab196495
Caspase 3	Affinity	AF6311
Cleaved Caspase 3	CST	9661
Cyto C	Abcam	ab110325
Complex IV	Invitrogen	PA5-26688
Drp1	Santa Cruz	sc-271583
p Drp1 S616	Affinity	AF8470
GSK3β	Selleck	F0142
p GSK3β S9	Selleck	F0299
HA tag	Proteintech	51064-2-AP
IRSI	Proteintech	17509-1-AP
p IRS1 mS302	Selleck	F1489
p IRSI hS307	CST	2381
p IRSI mS312	CST	2386
p IRSI hS616	Invitrogen	44-550G
LDHA	Affinity	DF6280
MFN1	Santa Cruz	sc-166644
MFN2	Santa Cruz	sc-515647
Complex I	Invitrogen	PA5-36493
NeuN	Servicebio	GB15138
OGG1/2	Santa Cruz	sc-376935
OPA1	Affinity	DF8587
PBX1	Invitrogen	PA5-17223
PDH e1-α	CST	3205
PDK1	Selleck	F1103
pPDK1 S241	Selleck	F0401
PDK4	Proteintech	83583-1-RR
Complex II	Affinity	DF12732
Complex III	Invitrogen	459140
β-Actin	Huabio	PSH03-63

### Immunoprecipitation assay

To assess the presence of TAT-PBX1 in brain tissue, immunoprecipitation was performed using the Anti-His Immunoprecipitation Kit (IK-1015, Biolinkedin, Shanghai, China), following the manufacturer’s instructions. Briefly, 50 mg of brain tissue was pulverized in liquid nitrogen and lysed in ice-cold RIPA buffer, supplemented with 1% phenylmethylsulfonyl fluoride and a protease inhibitor cocktail. The tissue homogenate was incubated on ice for 30 minutes and then centrifuged at 12,000 × *g* for 10 minutes at 4°C. The supernatant was collected, and total protein concentration was quantified and normalized using a BCA assay. An aliquot of the lysate was reserved as an input control. The remaining protein lysate was incubated with Anti-His magnetic beads at 4°C for 4 hours with gentle rotation. Mouse immunoglobulin G was used as a negative control. Following incubation, the magnetic beads were separated using a magnetic stand and washed twice with RIPA buffer. Beads were then resuspended in 1× SDS loading buffer and heated at 100°C for 10 minutes to elute the bound proteins. The supernatant was then collected for analysis. The denatured samples were subjected to Western blot analysis to detect the presence of His6-tagged proteins and PBX1.

### Transmission electron microscopy

The culture medium was carefully aspirated, and cells were gently washed twice with pre-chilled PBS. Cells were then fixed overnight at 4°C with pre-cooled 2.5% glutaraldehyde, ensuring full coverage of the cell monolayer. The following day, the fixative was removed, and cells were washed three times with PBS. Post-fixation was carried out in 1% osmium tetroxide for 1 hour at room temperature. After removing the osmium tetroxide and washing the cells three additional times with PBS, samples were dehydrated via a graded ethanol series and embedded in Durcupan acetamidomethyl resin. Ultrathin sections (60–80 nm) were cut using an ultramicrotome and collected on copper grids. Sections were counterstained with uranyl acetate and lead citrate. Ultrastructural analysis was performed using a transmission electron microscope (JEM-1200EX, JEOL, Tokyo, Japan), and images were acquired at magnifications of 4000× to 10,000× using a Veleta charge-coupled device camera (Olympus, Tokyo, Japan). Manual segmentation of cell and mitochondrial contours was conducted using the ImageJ software (version 1.53k, National Institutes of Health). Only mitochondria with clearly visible and intact inner membranes were included in subsequent quantitative analyses.

### Statistical analysis

All statistical analyses were performed using GraphPad Prism (version 8.0.3, San Diego, CA, USA) and R (version 4.5.0) software. For human studies, the Kolmogorov–Smirnov test was used to assess the normality of the data distribution, and Levene’s test was applied to evaluate the homogeneity of variance across groups. Data following a normal distribution are presented as mean ± standard deviation (SD), whereas non-normally distributed data are presented as medians and interquartile ranges. Categorical variables are reported as frequencies and percentages. For normally distributed data, Student’s *t*-test was used for between-group comparisons. For non-normally distributed data, the Mann–Whitney *U* test was used. The chi-square test was used for comparisons of categorical variables between groups. For cell and animal experiments, one-way analysis of variance (ANOVA) with the Holm–Sidak multiple-comparisons test was used for multi-group comparisons. For datasets that were non-normally distributed, the Kruskal–Wallis test with Dunn’s multiple-comparisons test was used. *Post hoc* comparisons were performed following a significant omnibus test. The association between MMSE scores and plasma PBX1 concentrations was analyzed using Spearman’s rank correlation analysis, with the corresponding *P*-values and correlation coefficients (ρ) calculated. The effects of two factors on the experimental outcomes were analyzed via repeated measures two-way ANOVA with Holm–Sidak correction for multiple comparisons. A binomial logistic regression model was also constructed to evaluate the association between PBX1 and MCI status while adjusting for covariates, reporting odds ratios (ORs) with 95% confidence intervals (CIs). A *P*-value of < 0.05 was considered to indicate statistical significance. All tests were two-sided. Additional methodological details are provided in **Additional file 1**.

## Results

### Binomial logistic regression evaluating the association between PBX1 and mild cognitive impairment (case-control analysis)

**Figure 1:**
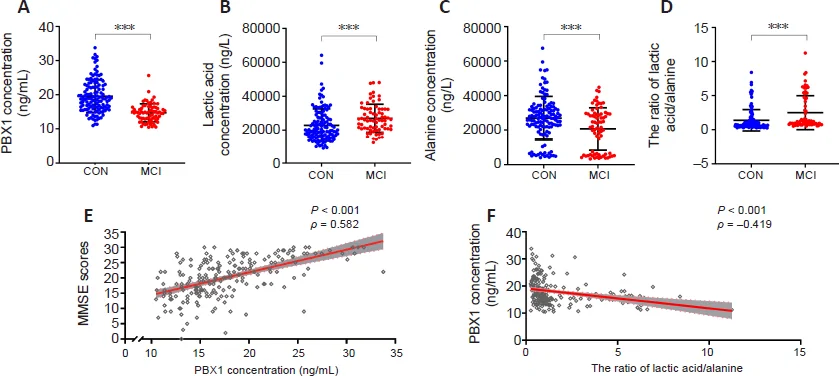
PBX1 plays a central role in the transition from MCI to AD. (A) Plasma PBX1 concentration. (B) Serum lactate concentration. (C) Serum alanine concentration. (D) The ratio of lactic acid concentration to alanine concentration. (E) Correlation analysis between plasma PBX1 concentration and MMSE score. (F) Correlation analysis between the lactate-to-alanine ratio and plasma PBX1 concentration. CON, *n* = 127; MCI, *n* = 73. Data are expressed as the median (interquartile range). Statistical analyses were performed using the unpaired Mann‒Whitney *U* test. The association between MMSE cognitive scores and plasma PBX1 concentrations was analyzed using Spearman’s rank correlation analysis, and the corresponding *P* value and correlation coefficient (ρ) were calculated. ***P < 0.001. AD: Alzheimer’s disease; CON: control group; MCI: mild cognitive impairment; MMSE: Mini-Mental State Examination; PBX1: pre-B-cell leukemia homeobox 1.

To determine the relationship between PBX1 and human cognitive function and metabolic status, we first conducted a case-control study to assess plasma PBX1 levels and key metabolite concentrations in CON subjects and individuals with MCI. The process for selecting study participants was as follows: Of the 200 potential subjects who were screened, all 200 met our eligibility criteria and were enrolled into the study (i.e., 73 with MCI and 127 CON). All 200 enrolled participants completed the study, with no attrition or missing data for any essential demographic, clinical, or laboratory variables. The baseline characteristics of the participants are summarized in **Table 1**. No significant differences were observed between the CON and MCI groups in terms of gender, body mass index, annual income, smoking status, or alcohol consumption history. However, participants in the MCI group were significantly older than those in the CON group. No significant differences in biochemical parameters (i.e., FBG, HbA1c, HDL-C, LDL-C, ALT, AST, LDHA, CK, CK-MB, total bilirubin, and serum creatinine levels) were detected between groups. These indicators were assessed to rule out the impact of glucose metabolism and liver or kidney disease on the results. Notably, compared with the CON group, the MCI group had significantly lower orientation, memory, attention, recall, verbal ability, and overall MMSE scores, which indicated impaired cognitive function. We observed that plasma PBX1 levels were significantly lower in the MCI group than in the control group (*P* < 0.001, **Figure 1A**). To assess the independence of this association, we performed binomial logistic regression analyses (**Table 2**). The inverse association between MCI status and lower PBX1 levels remained significant after adjusting for demographic factors alone (*P* < 0.001, Model 2: age and gender) and upon further adjustment for cardiometabolic and social factors (*P* < 0.001, Model 3: age, gender, TC, TG, and marital status). These results indicated that the association was robust and independent of the known confounding factors.

**Table 1:** Characteristics of participants

Demographic variable	Control (*n* = 127)	MCI (*n* = 73)	*P*
Age (yr)	71.0 (69.0, 75.0)	75.0 (71.0, 79.0)	< 0.001
Gender			0.101
Male	71 (55.9)	32 (43.8)	
Female	56 (44.1)	41 (56.2)	
BMI (kg/m^2^)	23.7 (22.2, 26.5)	24.0 (21.7, 25.9)	0.244
FBG (mM)	5.54 (5.28, 5.88)	5.60 (5.36, 5.92)	0.233
HbA1c	5.60 (5.40, 5.80)	5.70 (5.50, 5.90)	0.445
Marital status			0.005
Single/divorced/widowed	64 (50.4)	52 (71.2)	
Married	63 (49.6)	21 (28.8)	
Education level			0.036
Illiterate	3 (2.36)	7 (9.59)	
Primary	85 (66.9)	50 (68.5)	
Secondary and higher	39 (30.7)	16 (21.9)	
Family income (yr)			0.157
≤ 1000 CNY	20 (15.7)	19 (26.0)	
1001–3000 CNY	67 (52.8)	36 (49.3)	
3001–5000 CNY	10 (7.87)	9 (12.3)	
5001–8000 CNY	14 (11.0)	4 (5.48)	
> 8000 CNY	16 (12.6)	5 (6.85)	
Smoking status			0.131
Smoker	38 (29.9)	14 (19.2)	
Non-smoker	89 (70.1)	59 (80.8)	
Drinking status			0.208
Drinker	45 (35.4)	19 (26.0)	
Non-drinker	82 (64.6)	54 (74.0)	
Temporal orientation (point)	5.00 (3.00, 5.00)	1.00 (0.00, 3.00)	< 0.001
Spatial orientation (point)	5.00 (5.00, 5.00)	5.00 (2.00, 5.00)	< 0.001
Registration (point)	3.00 (3.00, 3.00)	3.00 (1.00, 3.00)	< 0.001
Attention and calculation (point)	3.00 (1.00, 5.00)	0.00 (0.00, 1.00)	< 0.001
Recall (point)	2.00 (1.00, 3.00)	1.00 (0.00, 2.00)	< 0.001
Language (point)	8.00 (7.00, 9.00)	5.00 (4.00, 7.00)	< 0.001
MMSE total score (point)	24.0 (21.0, 27.0)	14.0 (12.0, 17.0)	< 0.001
Total cholesterol (mM)	5.04 (4.70, 5.76)	5.48 (4.99, 6.00)	0.006
Triglyceride (mM)	0.98 (0.78, 1.35)	1.33 (0.85, 1.72)	0.004
HDL-C (mM)	1.37 (1.19, 1.58)	1.39 (1.10, 1.58)	0.532
LDL-C (mM)	2.99 ± 0.76	3.20 ± 0.74	0.056
ALT (U/L)	16.0 (13.0, 23.0)	16.0 (12.0, 22.0)	0.601
AST (U/L)	19.0 (16.0, 24.0)	19.0 (16.0, 23.0)	0.412
LDH (U/L)	228 (206, 249)	228 (204, 246)	0.431
CK (U/L)	100 (73.0, 139)	92.0 (66.0, 139)	0.430
CK-MB (ng/L)	12.0 (10.0, 15.0)	12.0 (9.00, 13.0)	0.115
Total bilirubin (μM)	14.6 (12.1, 17.8)	13.9 (11.1, 18.4)	0.232
Creatinine (μM)	66.0 (58.0, 76.0)	63.0 (53.0, 76.0)	0.489

For categorical variables, data are presented as frequencies (percentages); for normally distributed data, as the mean ± standard deviation (mean ± SD); and for non-normally distributed data, as the median (interquartile range). For normally distributed data, Student’s t-test was used for between-group comparisons; otherwise, the Mann–Whitney U test was applied. The chi-square test was used for comparisons of categorical variables between groups. ALT: Alanine aminotransferase; AST: aspartate transaminase; BMI: body mass index; CK: creatine kinase; CK-MB: creatine kinase MB; CNY: Chinese Yuan; FBG: fasting blood glucose; HbA1c: glycosylated hemoglobin; HDL-C: high-density lipoprotein cholesterol; IQR: interquartile range; LDH: lactate dehydrogenase; LDL-C: low-density lipoprotein cholesterol; MCI: mild cognitive impairment; MMSE: Mini-Mental State Examination.

**Table 2:** Binomial logistic regression evaluating the association of plasma PBX1 with MCI

Model	Factor	*β*	Wald	SE	OR (95%CI)	P
Model 1	Intercept	6.134	33.44	1.125	–	< 0.001
	PBX1	–0.409	29.721	0.071	0.665 (0.579–0.763)	< 0.001
Model 2	Intercept	–2.229	0.581	2.925	–	0.446
	PBX1	–0.425	32.134	0.075	0.653 (0.564–0.757)	< 0.001
	Age	0.114	8.516	0.039	1.121 (1.038–1.210)	0.004
	Gender (female)	0.570	2.542	0.358	1.769 (0.877–3.567)	0.111
Model 3	Intercept	–5.128	2.579	3.193	–	0.108
	PBX1	–0.417	29.24	0.077	0.659 (0.567–0.767)	< 0.001
	Age	0.122	8.733	0.041	1.130 (1.039–1.230)	0.003
	Gender (female)	0.142	0.129	0.395	1.152 (0.532–2.499)	0.719
	Marital status (single/divorced/widowed)	0.913	5.512	0.389	2.492 (1.163–5.342)	0.019
	TC	0.241	1.386	0.205	1.272 (0.852–1.900)	0.239
	TG	0.425	2.082	0.295	1.530 (0.859–2.726)	0.149

Model 1: Unadjusted; Model 2: adjusted for age and gender; Model 3: adjusted for age, gender, marital status, TC, and TG. MCI: Mild cognitive impairment; PBX1: Pre-B-cell leukemia transcription factor 1; TC: total cholesterol; TG: triglyceride.

Lactic acid is the final product of glycolysis, whereas alanine is generated via pyruvate transamination, which demonstrates the amount of available pyruvate. The concentrations of lactic acid and alanine, along with their ratio, serve as critical biomarkers for assessing cellular energy metabolic status, which specifically indicates the balance between glycolytic flux and OXPHOS efficiency (Vaz et al., 2012; Sharma et al., 2021; Shayota, 2024). We found that the MCI group exhibited significantly higher blood lactic acid levels and reduced alanine concentrations compared with the CON group (*P* < 0.001, **Figure 1B** and **C**). Moreover, the lactic acid to alanine ratio was significantly higher in the MCI group than in the CON group (*P* < 0.001, **Figure 1D**). These results indicate a possible dysregulation of central nervous system metabolism. Spearman’s rank correlation analysis indicated a significant moderate positive correlation between plasma PBX1 levels and MMSE scores (*P* < 0.001, **Figure 1E**), as well as a significant moderate negative correlation between plasma PBX1 levels and lactic acid to alanine ratio (*P* < 0.001, **Figure 1F**). These findings suggest an imbalance in cerebral energy homeostasis in patients with MCI and indicate that PBX1 is a key regulator of the progression from MCI to AD.

### TAT-PBX1 reduces amyloid-β oligomers-induced apoptosis in SH-SY5Y cells

**Figure 2:**
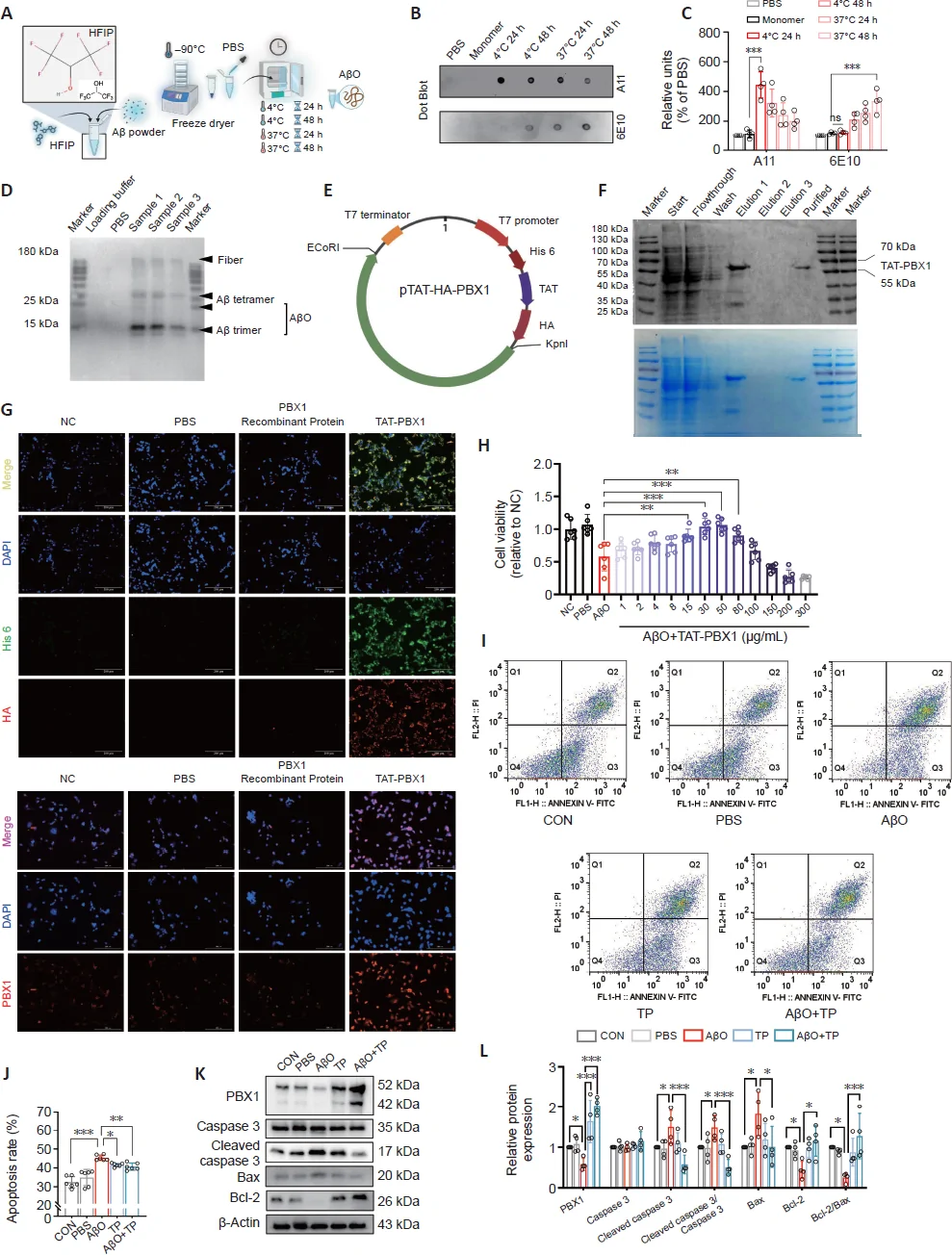
TAT-PBX1 reduces AβO-induced apoptosis in SH-SY5Y cells. (A) Procedure for incubating Aβ at different temperatures. Created with BioRender.com. (B) Representative dot blot showing A11 and 6E10-positive Aβ aggregates prepared under different conditions (*n* = 4). (C) Quantification of the dot blot results. (D) Western blot analysis of the Aβ molecular weight distribution in whole-cell lysates after the incubation of AβOs with SH-SY5Y cells for 24 hours. (E) Schematic diagram of the TAT-PBX1 plasmid: the PBX1 DNA sequence was inserted into the pTAT-HA plasmid vector at the C-terminus of the TAT peptide, and contains an N-terminal His 6 tag for purification and an HA tag for distinguishing TAT from PBX1. (F) Detection of the affinity-purified TAT-PBX1 fusion protein by SDS‒PAGE and Coomassie brilliant blue staining. (G) Immunofluorescence staining of SH-SY5Y cells under different treatment conditions used His 6 (in green) and HA (in red) antibodies to evaluate TAT-PBX1 internalization (scale bar: 200 μm). Subsequent PBX1 antibody (in red) staining under varied conditions assessed the ability of TAT-PBX1 to enhance endogenous PBX1 expression in SH-SY5Y cells (scale bar: 200 μm). (H) CCK-8 assay measuring cell viability after coincubation with different concentrations of AβOs and TAT-PBX1, normalized to the NC group (*n* = 6). (I) Representative flow cytometry scatter plots from Annexin V/PI double staining showing apoptosis levels in SH-SY5Y cells co-incubated with AβOs (*n* = 3). (J) Quantification of the Annexin V/PI apoptosis assay (*n* = 3). (K) Representative western blot bands for PBX1, Caspase 3, Cleaved Caspase 3, Bax, and Bcl-2 after AβO exposure in SH-SY5Y cells (*n* = 4). (L) Quantitative analysis of western blot data for PBX1, Caspase 3, Cleaved Caspase 3, Bax, and Bcl-2 levels (*n* = 4). Data are expressed as the mean ± SD. Group differences were analyzed using one-way analysis of variance (ANOVA), followed by Holm–Sidak multiple-comparisons tests. *Post hoc* comparisons were performed only after a significant omnibus ANOVA.*P < 0.05, **P < 0.01, ***P < 0.001. Aβ: Amyloid-beta; AβO: amyloid beta oligomer; CON: control; NC: normal control; PBS: phosphate buffered saline; PBX1: pre-B-cell leukemia homeobox 1; PI: propidium iodide; SDS‒PAGE: sodium dodecyl sulfate polyacrylamide gel electrophoresis; TAT: trans-activator of transcription peptide: TP: TAT-PBX1.

Aβ_1–42_, an amphipathic peptide, easily forms toxic oligomers when investigated *in vitro*. We subjected Aβ_1–42_ to four different conditions (4°C for 24 hours, 4°C for 48 hours, 37°C for 24 hours, and 37°C for 48 hours) to determine the optimal conditions for clumping together. We also examined its toxicity toward SH-SY5Y cells (**Figure 2A**). A dot blot analysis was used to determine aggregation of Aβ_1–42_, which was first dissolved in hexafluoroisopropanol, followed by incubation under the abovementioned conditions. PBS was used as the negative control. Samples incubated at 4°C for 24 hours showed the strongest A11-positive signal, which is a sign of the presence of oligomers. Samples incubated at 37°C for 48 hours showed the strongest 6E10 signal (*P* < 0.001, **Figure 2B** and **C**). To assess the neurotoxicity of AβO samples prepared under the various conditions, SH-SY5Y cells were subjected to AβO at different concentrations (i.e., 0, 0.75, 1.5, 3, 6, 12, 25, 50, 100, 150, and 200 μM) for 24 hours. The Cell Counting Kit-8 (CCK-8) assays demonstrated that AβO diminished SH-SY5Y cell viability in a dose-dependent manner (**Additional Figure 2A–D**). Therefore, we chose AβO samples that had been incubated at 50 μM (4°C for 24 hours), 100 μM (4°C for 48 hours), 100 μM (37°C for 24 hours), and 50 μM (37°C for 48 hours) for subsequent sets of experiments. Furthermore, Western blot analysis was conducted to determine which incubation condition produced AβO species with the most pronounced neurotoxicity. Samples incubated at 4°C for 24 hours showed marked increases in Cyto C, cleaved caspase-3, and the cleaved caspase-3/caspase-3 ratio, indicating that apoptosis was strongly induced (**Additional Figure 2E** and **F**). Thus, this condition (i.e., 4°C for 24 hours) was selected for the preparation of AβO in all subsequent studies. After the preformed aggregates were added to the SH-SY5Y cell culture medium, oligomers of 15–30 kDa were detected in the cell lysates after 24 hours, which indicated that the prepared AβO samples can be internalized by cells (**Figure 2D**).

Next, a prokaryotic expression vector encoding the TAT-PBX1 fusion protein was successfully constructed (**Figure 2E**). The full-length PBX1 DNA sequence was fused downstream of the TAT peptide sequence in the pTAT-HA vector. This vector has an N-terminal His6 tag for purification and an HA tag to differentiate the TAT part from the PBX1 part. SDS-PAGE showed a band at approximately 55 kDa, which was consistent with our expectations given the molecular weight of the TAT-PBX1 fusion protein (**Figure 2F**). Immunofluorescence staining showed that TAT-PBX1 was effectively taken up by cells, as shown by the positive staining for both the His6 and HA tags. In contrast, the control PBX1 protein without the TAT domain showed limited uptake (**Figure 2G**).

SH-SY5Y cells were treated with varying concentrations of TAT-PBX1 to determine its effect on AβO-induced apoptosis. TAT-PBX1 was administered to SH-SY5Y cells after a 24-hour exposure to 50 μM AβO. CCK-8 assays demonstrated that TAT-PBX1 (at concentrations of 15, 30, 50, and 80 μg/mL) increased cell viability significantly more than AβO treatment alone (*P* < 0.01, **Figure 2H**). Flow cytometry analysis using Annexin V/propidium iodide double staining showed that TAT-PBX1 mitigated AβO-induced apoptosis (*P* < 0.01, **Figure 2I** and **J**). These findings were further corroborated by Western blot analysis of the apoptotic markers (*P* < 0.05, **Figure 2K** and **L**). Collectively, these results indicated that our prepared TAT-PBX1 can be internalized by cells and reduce AβO-induced neuronal apoptosis.

### TAT-PBX1 reverses amyloid-β oligomers-induced impairment of the insulin signaling pathway

**Figure 3:**
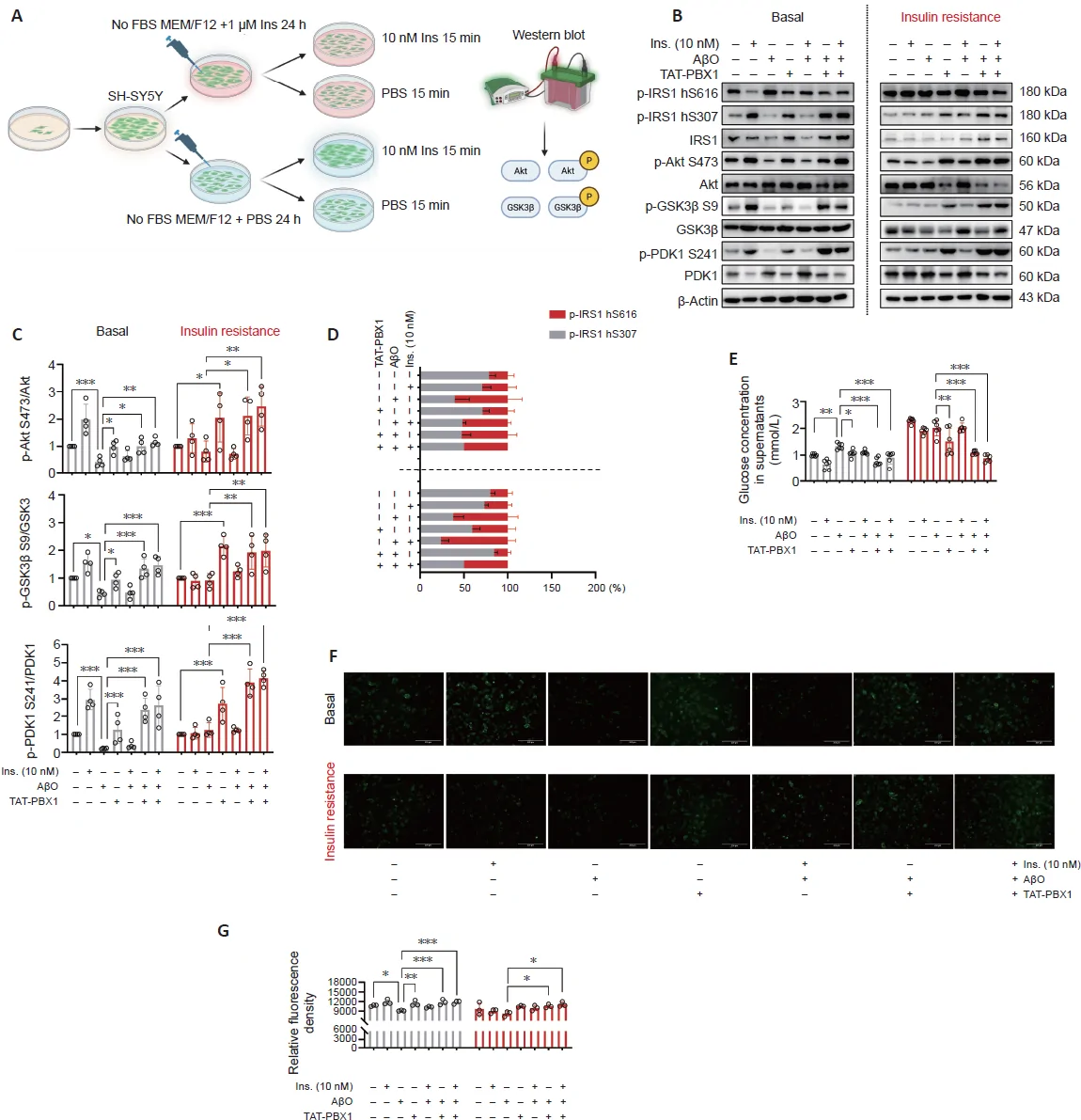
TAT-PBX1 restores insulin signaling upon the AβO intervention. (A) Schematic diagram of the establishment of the insulin resistance model in SH-SY5Y cells. Created with BioRender.com. (B) Representative western blot bands showing the effects of insulin, AβO, and/or TAT-PBX1 treatment under basal and insulin-resistant conditions on the levels of IRS1 phosphorylated at Ser616 (p-IRS1 hS616), IRS1 phosphorylated at Ser307 (p-IRS1 hS307), total IRS1, Akt phosphorylated at Ser473 (p-Akt S473), total Akt, GSK3β phosphorylated at Ser9 (p-GSK3β S9), total GSK3β, PDK1 phosphorylated at Ser241 (p-PDK1 S241), and total PDK1 (*n* = 4). (C) Quantification of the expression ratios of p-Akt S473/Akt, p-GSK3β S9/GSK3β, and p-PDK1 S241/PDK1 (*n* = 4). (D) Proportions of p-IRS1 hS616 and p-IRS1 hS307 relative to total IRS1 (*n* = 4). (E) Glucose concentration in the culture medium under basal and insulin-resistant states after treatment with insulin, AβO, and/or TAT-PBX1 (*n* = 6). (F) Representative fluorescence images of 2-Deoxy-2-[(7-nitro-2,1,3-benzoxadiazol-4-yl)amino]-D-glucose (2-NBDG)-labeled glucose uptake in SH-SY5Y cells under basal and insulin-resistant conditions, scale bars: 200 μm. (G) Quantification of the relative fluorescence intensity from the 2-NBDG glucose uptake assay (*n* = 3). Data are expressed as the mean ± SD. Group differences were analyzed using one-way analysis of variance (ANOVA), followed by the Holm–Sidak multiple-comparisons test. Post hoc comparisons were performed only after a significant omnibus ANOVA. *P < 0.05, **P < 0.01, and ***P < 0.001. 2-NBDG: 2-Deoxy-2-[(7-nitro-2,1,3-benzoxadiazol-4-yl) mino]-D-glucose; AβO: amyloid beta oligomer; GSK3β: glycogen synthase kinase 3 beta; Ins: insulin; IRS1: insulin receptor substrate 1; PDK1: 3-phosphoinositide-dependent protein kinase-1; TAT-PBX1: trans-activator of transcription peptide-pre-B-cell leukemia homeobox 1.

To better understand whether TAT-PBX1 restores insulin sensitivity by modulating insulin signaling in neurons under AβO treatment, we first established an insulin resistance model in SH-SY5Y cells (**Figure 3A**). Compared with chronic insulin treatment (1 μM at 24 hours), acute insulin stimulation (10 nM at 15 minutes) in the control group significantly increased p-Akt S473/Akt and p-GSK3β S9/GSK3β ratios, confirming successful establishment of the insulin resistance model (**Additional Figure 3A** and **B**). As noted earlier, abnormal phosphorylation of IRS1, a key molecule in the insulin signaling cascade, is among the core mechanisms of insulin resistance. Phosphorylation of IRS1 at hS307 (mS302 in mice) is essential for effective insulin signal transduction (Danielsson et al., 2005). Therefore, the p-IRS1 hS307 to p-IRS1 hS616 ratio (corresponding to the p-IRS1 mS302 to mS612 ratio in mice) serves as an indicator of the insulin responsiveness of IRS1. Additionally, 3-phosphoinositide-dependent protein kinase-1 (PDK1) directly activates Akt via phosphorylation, whereas GSK3β functions as a downstream target of Akt, the activity of which is suppressed by Akt-mediated phosphorylation (Wang et al., 2025). This signaling cascade represents a core mechanism for insulin-mediated anabolic activity and glucose homeostasis. Under basal conditions, AβO exposure inhibited insulin signaling and reduced glucose uptake, which were not observed in the untreated group. These changes were reflected in a significant increase in the p-IRS1 hS616 level and a decrease in the p-IRS1 hS302 level, alongside marked reductions in the p-Akt S473/Akt, p-GSK3β S9/GSK3β, and p-PDK1 S241/PDK1 ratios (*P* < 0.01, **Figure 3B** and **C** and **Additional Figure 4**). Notably, TAT-PBX1 treatment under basal conditions reversed the AβO-induced insulin signaling impairment and AβO-induced insulin resistance in SH-SY5Y cells (*P* < 0.05, **Figure 3C**). These changes also paralleled a significant decrease in the p-IRS1 hS616/IRS1 ratio and an increase in the p-IRS1 hS302/IRS1 ratio (**Figure 3D**). Furthermore, under insulin resistance conditions, TAT-PBX1 treatment restored glucose uptake, as indicated by an increase in 2-(N-(7-Nitrobenz-2-oxa-1,3-diazol-4-yl) Amino)-2-Deoxyglucose (2-NBDG) fluorescence intensity and a decrease in glucose concentration in the supernatant (*P* < 0.05, **Figure 3E–G**). Collectively, these findings indicate that TAT-PBX1 increases insulin sensitivity in SH-SY5Y cells under both basal and insulin-resistant conditions.

### TAT-PBX1 restores cellular energy homeostasis upon treatment with amyloid-β oligomers

**Figure 4:**
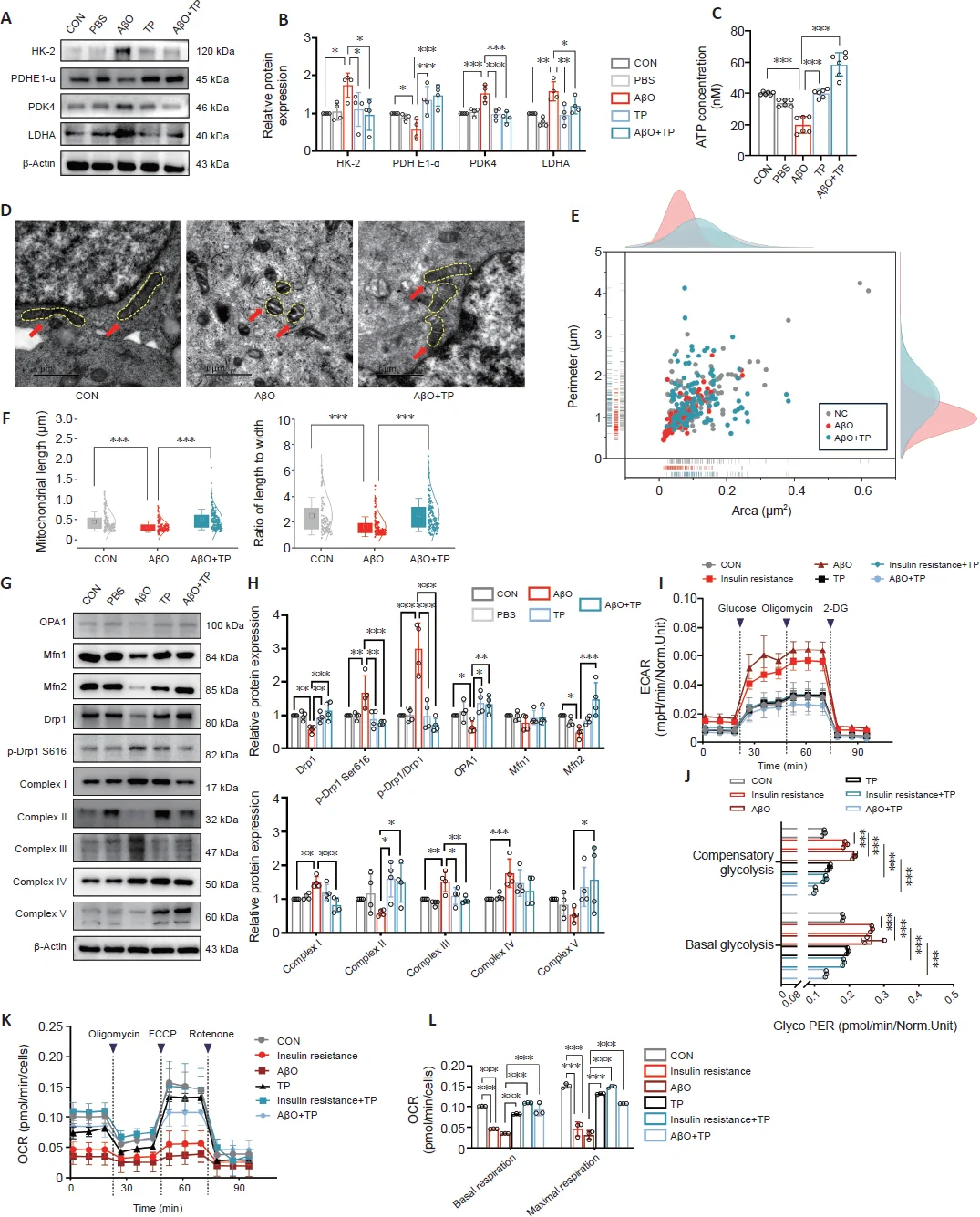
TAT-PBX1 improves mitochondrial function and promotes OXPHOS in response to the AβO intervention. (A) Representative western blot bands showing the expression levels of HK-2, PDH E1-α, PDK4, and LDHA in SH-SY5Y cells co-incubated with AβOs (*n* = 4). (B) Quantitative analysis of HK-2, PDH E1-α, PDK4, and LDHA protein expression (*n* = 4). (C) Intracellular ATP concentrations in SH-SY5Y cells after AβO treatment (*n* = 6). (D) TEM images showing the mitochondrial morphology (scale bar: 1 μm). (E) Scatter and frequency distribution plots of the mitochondrial perimeter and area. (F) Quantitative analysis of the mitochondrial length and aspect ratio. (G) Representative western blot bands for mitochondrial dynamics proteins OPA1, Mfn1, Mfn2, Drp1, Drp1 phosphorylated at Ser616 (p-Drp1 S616), and OXPHOS complexes I–V (*n* = 4). (H) Quantification of the protein levels in G (*n* = 4). (I) ECAR measured using an Agilent Seahorse XF analyzer (*n* = 3). (J) Proton efflux rate during basal and compensatory glycolysis (*n* = 3). (K) OCR measured using an Agilent Seahorse XF analyzer (*n* = 3). (L) OCR during basal and maximal respiration (*n* = 3). Data are expressed as the mean ± SD. Group differences were analyzed using one-way analysis of variance (ANOVA), followed by the Holm–Sidak multiple-comparisons test. *Post hoc* comparisons were performed only after a significant omnibus ANOVA. *P < 0.05, **P < 0.01, ***P < 0.001. ATP: Adenosine triphosphate; AβO: amyloid beta oligomer; CON: control; Drp1: dynamin-related protein 1; ECAR: extracellular acidification rate; HK-2: hexokinase 2; LDHA: lactate dehydrogenase A; Mfn1: mitofusin 1; Mfn2: mitofusin 2; OCR: oxygen consumption rate; OPA1: optic atrophy 1; OXPHOS: oxidative phosphorylation; PBS: phosphate buffered saline; PDH: pyruvate dehydrogenase; PDK4: pyruvate dehydrogenase kinase 4; TEM: transmission electron microscopy.

We then examined the impact of TAT-PBX1-mediated restoration of insulin sensitivity on downstream metabolic patterns. PDKs inhibit OXPHOS by deactivating the pyruvate dehydrogenase (PDH) E1-α subunit. Therefore, we evaluated protein expressions at key glycolysis and OXPHOS nodes. Western blot analysis revealed that compared with AβO treatment, TAT-PBX1 treatment induced a significantly greater reduction in PDK4 protein levels (*P* < 0.05, **Figure 4A** and **B**). Importantly, TAT-PBX1 also downregulated the expression of hexokinase-2 (HK-2) and LDHA while increasing PDH E1-α expression and ATP production (*P* < 0.001, **Figure 4C**). TAT-PBX1 suppresses glycolytic proteins PDK4, HK-2, and LDHA, and upregulates PDH E1-α, which indicates its potential to positively regulate OXPHOS. We then examined whether the ATP increase was driven by enhanced mitochondrial oxidative metabolism.

Because efficient mitochondrial oxidative metabolism relies on proper ultrastructure and dynamics, we examined mitochondrial morphology and dynamics-related protein expression via transmission electron microscopy (TEM) and Western blot analysis. TEM revealed that, in contrast to the control treatment, AβO treatment induced mitochondrial swelling, which was accompanied by an elongated shape, shortened and disorganized cristae, and reduced mitochondrial volume (**Figure 4D**). In contrast, TAT-PBX1 treatment restored normal mitochondrial morphology, characterized by well-organized cristae (**Figure 4D**), larger and more numerous mitochondria (**Figure 4E**), and an elevated mitochondrial aspect ratio (**Figure 4F**), which indicated improved structural integrity and potentially enhanced energy metabolism. Consistent with the TEM findings, AβO treatment significantly increased the phosphorylated dynamin-related protein 1 (p-Drp1) S616/Drp1 ratio and reduced the expression of fusion-related proteins, optic atrophy 1 (OPA1) and mitofusin 1/2 (Mfn1/2), which indicated impaired mitochondrial dynamics (*P* < 0.01, **Figure 4G** and **H**). In contrast, TAT-PBX1 treatment decreased the p-Drp1 S616/Drp1 ratio, restoring mitochondrial dynamics homeostasis.

Mitochondrial electron transport chain (ETC) complexes (complexes I–V) form supercomplexes that couple electron transfer to proton pumping, creating a gradient that powers ATP synthesis via complex V and maintains OXPHOS efficiency. Therefore, we investigated the impact of AβO and TAT-PBX1 treatment on ETC complexes. Exposure to AβO significantly elevated the expression of respiratory complexes I, III, and IV, which suggested possible metabolic dysregulation (*P* < 0.05, **Figure 4G** and **H**). In contrast, TAT-PBX1 lowered the levels of respiratory complexes I, II, III, and V. However, to comprehensively examine the metabolic state of a cell, changes in the levels of complex I–V proteins must be determined, alongside respiration rate. Subsequently, we measured how much glycolysis and OXPHOS contribute to making ATP in cells. The ECAR was significantly higher in SH-SY5Y cells that were treated with either insulin resistance or AβO, which suggested that the cells were using more glycolytic metabolism (*P* < 0.001, **Figure 4I** and **J**). TAT-PBX1 treatment decreased the ECAR in both scenarios, which indicated inhibition of glycolysis. Given that the ECAR shows acidification from both glycolysis and the TCA cycle, we also measured the OCR to determine the effectiveness of mitochondrial function (Schmidt et al., 2021). OCR analysis indicated that insulin resistance and AβO exposure diminished basal respiration, maximal respiration, ATP production, and proton leakage, and TAT-PBX1 treatment rescued these impairments (*P* < 0.001, **Figure 4K** and **L**). These findings indicate that TAT-PBX1 possesses significant potential to alter cellular energy metabolism. By simultaneously enhancing the expression and function of mitochondrial ETC complexes and suppressing glycolysis, TAT-PBX1 shifts cells from a glycolysis-dependent state to an efficient OXPHOS state, thereby fundamentally restoring energy homeostasis.

### PBX1 binds to the insulin receptor substrate 1 promoter to facilitate its transcription and regulate energy metabolism

**Figure 5:**
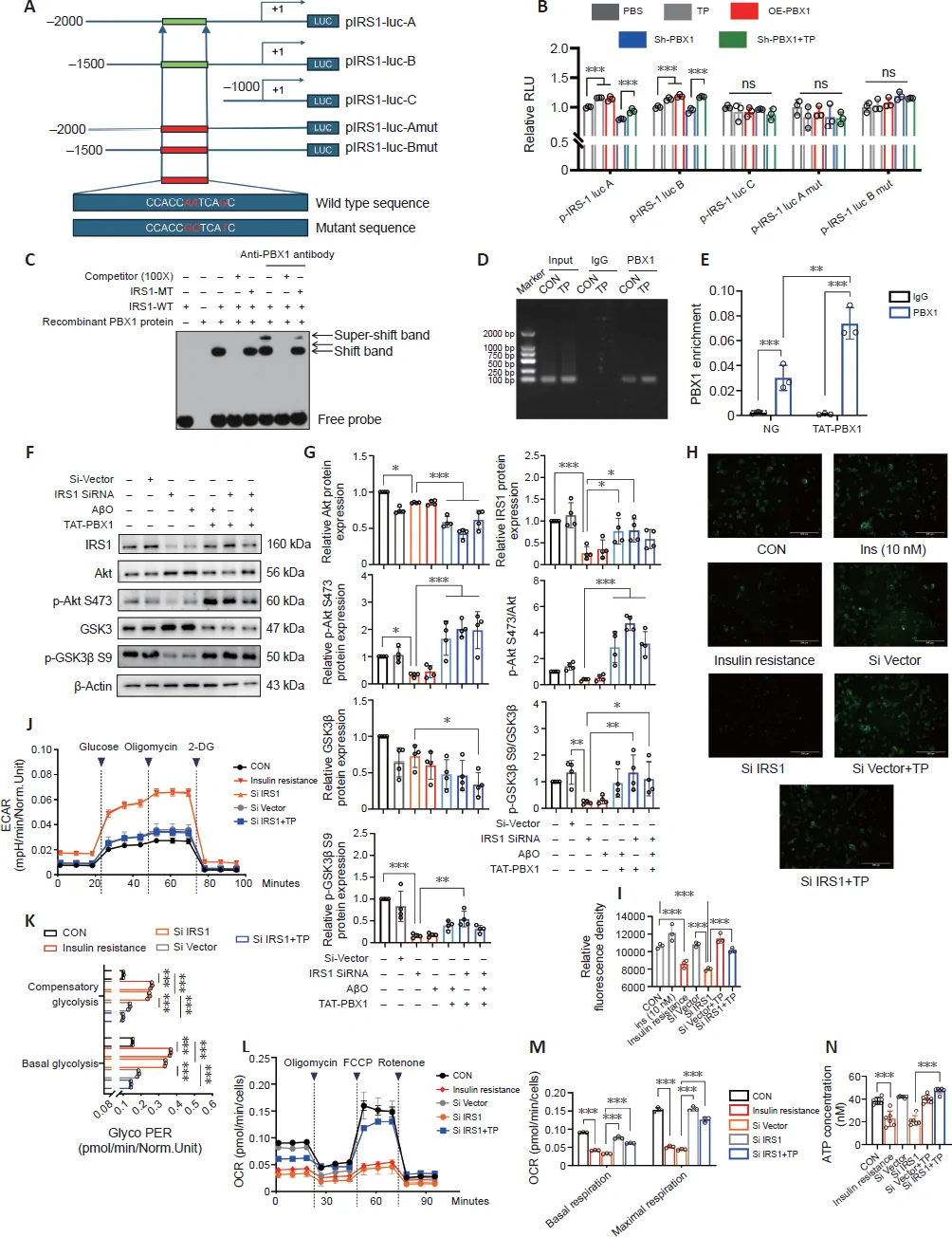
PBX1 binds to the IRS1 promoter to promote its transcription and regulate energy metabolism. (A) Schematic representation of human IRS1 promoter truncations and site-directed mutagenesis constructs; arrows indicate transcription start sites. (B) Luciferase activity in SH-SY5Y cells transfected with various IRS1 promoter constructs and treated with TAT-PBX1, PBX1 overexpression, PBX1 knockdown, or PBX1 knockdown plus TAT-PBX1 (*n* = 3). (C) EMSA image demonstrating PBX1 binding to the IRS1 promoter region *in vitro*. (D) Gel image showing IRS1 promoter DNA enrichment through PBX1 chromatin immunoprecipitation in SH-SY5Y cells treated with or without TAT-PBX1. (E) Quantitative PCR results of IRS1 promoter enrichment from PBX1-immunoprecipitated chromatin (*n* = 3). (F) Representative Western blot bands illustrating the protein levels of p-Akt S473, Akt, p-GSK3β S9, and GSK3β in SH-SY5Y cells following IRS1 silencing (*n* = 4). (G) Quantification of protein levels (*n* = 4). (H) Representative images of 2-NBDG-labeled SH-SY5Y cells, scale bars: 200 μm. (I) Quantification of relative fluorescence intensity from the 2-NBDG glucose uptake assay (*n* = 3). (J) ECAR measurements in SH-SY5Y cells under IRS1-silenced and insulin-resistant conditions (*n* = 3). (K) Proton efflux rate during basal and compensatory glycolysis following IRS1 silencing and insulin resistance (*n* = 3). (L) OCR measurements in SH-SY5Y cells with IRS1 silencing and insulin resistance (*n* = 3). (M) OCR during basal and maximal respiration under the same conditions (*n* = 3). (N) ATP concentration in SH-SY5Y cells after IRS1 knockdown and/or TAT-PBX1 treatment (*n* = 6). Data are expressed as the mean ± SD. Data are expressed as the mean ± SD. Group differences were analyzed using one-way analysis of variance (ANOVA), followed by the Holm–Sidak multiple-comparisons test. *Post hoc* comparisons were performed only after a significant omnibus ANOVA. *P < 0.05, **P < 0.01, ***P < 0.001. 2-NBDG: 2-Deoxy-2-[(7-nitro-2,1,3-benzoxadiazol-4-yl) amino]-D-glucose; ATP: adenosine triphosphate; ChIP: chromatin immunoprecipitation; ECAR: extracellular acidification rate; EMSA: electrophoretic mobility shift assay; GSK3β: glycogen synthase kinase 3 beta; Ins: insulin; IRS1: insulin receptor substrate 1; OCR: oxygen consumption rate; PBX1: pre-B-cell leukemia homeobox 1; PCR: polymerase chain reaction; TP: TAT-PBX1.

To clarify the mechanism by which TAT-PBX1 enhances insulin sensitivity in SH-SY5Y cells, we initially constructed a complete IRS1 promoter (2000 bp, referred to as pIRS1-luc-A) and subsequently cloned it into the pGL4.19 vector (**Figure 5A**). We performed dual-luciferase reporter assays using four different methods: TAT-PBX1 treatment, PBX1 overexpression, PBX1 knockdown, and PBX1 knockdown with TAT-PBX1 treatment. We found that pIRS1-luc-A promoter activity increased markedly when TAT-PBX1 was used and PBX1 was overexpressed (*P* < 0.001, **Figure 5B**). In contrast, knocking down PBX1 ceased promoter activity, which was restored by TAT-PBX1 (*P* < 0.001, **Figure 5B**), suggesting that PBX1 plays a positive role in activating IRS1 transcription. We then generated truncated promoter constructs, pIRS1-luc-B (–1500 bp to +1 bp) and pIRS1-luc-C (–1000 bp to +1 bp) and cloned them into the pGL4.19 vector to further narrow down the PBX1-responsive element. These constructs were simultaneously transfected with a herpes simplex virus-thymidine kinase promoter into SH-SY5Y cells. TAT-PBX1 and PBX1 overexpression significantly enhanced pIRS1-luc-B activity while exerting no influence on pIRS1-luc-C activity (**Figure 5B**). Similarly, knocking down PBX1 decreased pIRS1-luc-B activity, which was restored by TAT-PBX1 treatment, while pIRS1-luc-C activity remained the same. Taken together, these findings indicate that the PBX1 binding site resides within the –1500 to –1000 bp region of the IRS1 promoter.

Analysis of the JASPAR database identified a possible PBX1 binding motif between −1468 and –1456 bp. The electrophoretic mobility shift assay results demonstrated specific binding between the PBX1 protein and the WT IRS1 probe, which was accompanied by a supershift in the presence of the PBX1 antibody. This binding was stopped by a 100× molar excess of the unlabeled competitor probe (**Figure 5C**). Chromatin immunoprecipitation assays confirmed the *in vivo* interaction: compared with the control treatment, TAT-PBX1 treatment induced significantly greater PBX1 enrichment at the IRS1 promoter (*P* < 0.01, **Figure 5D** and **E**), which indicated enhanced genomic recruitment. Mutant promoters (i.e., pIRS1-luc-A-mut/pIRS1-luc-B-mut) with disrupted PBX1 motifs lost responsiveness to TAT-PBX1 and PBX1 overexpression (*P* > 0.05, **Figure 5B**), as indicated by the lack of statistically significant differences in relative light units among the components, confirming the functional dependence on this site.

We next examined the effects of IRS1 silencing on energy metabolism. Western blot analysis demonstrated that silencing IRS1 expression significantly disrupted insulin signaling, as indicated by the decreased phosphorylation of Akt and GSK3β, which paralleled the effects of AβO exposure (*P* < 0.05, **Figure 5F** and **G**). This suggested that insulin signaling was compromised. Notably, cotreatment with TAT-PBX1 reinstated IRS1 expression and normalized the p-Akt S473/Akt and p-GSK3β S9/GSK3β ratios (*P* < 0.05, **Figure 5G**). Silencing IRS1 decreased glucose uptake, as indicated by a decrease in 2-NBDG fluorescence intensity, which was counterbalanced by TAT-PBX1 (*P* < 0.05, **Figure 5H** and **I**). The extracellular flux analysis demonstrated that, compared with the control treatment, insulin resistance treatment or IRS1 silencing produced a significantly greater elevation of the ECAR while diminishing basal respiration (*P* < 0.001, **Figure 5J–M**), maximal respiration, proton leakage, and ATP production (*P* < 0.001, **Figure 5N**). TAT-PBX1 treatment significantly reversed these metabolic changes by lowering the ECAR and raising OXPHOS parameters in cells that had been treated with IR or silenced IRS1. These results indicate that PBX1 directly interacts with the IRS1 promoter at –1468/–1456 bp to facilitate transcription, thereby reinstating insulin sensitivity and energy homeostasis in response to AβO-induced stress.

### TAT-PBX1 improves memory and reduces neuronal APP/PS1 transgenic mice

**Figure 6:**
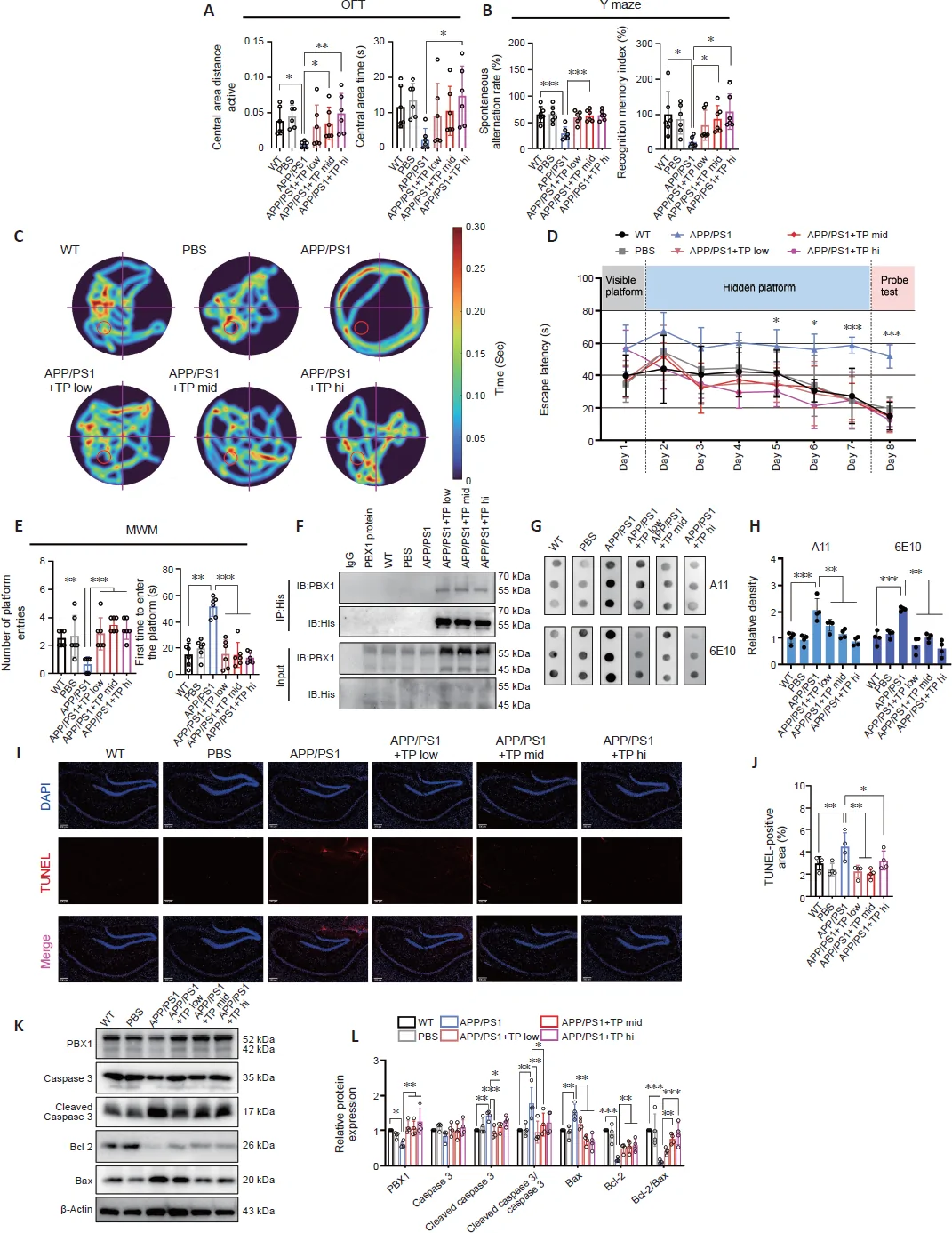
TAT-PBX1 improves memory and reduces neuronal apoptosis in APP/PS1 mice. (A) Percentage of time spent and distance traveled in the center area in the open field test (*n* = 6). (B) Spontaneous alternation percentage and recognition memory index in the Y-maze test (*n* = 6). (C) Representative heatmaps of movement trajectories in the Morris Water Maze test, colors on the heatmap represent dwell time, with red indicating longer and blue indicating shorter durations. (D) Escape latency during the visible platform phase, hidden platform phase, and probe test (*n* = 6). (E) Number of platform crossings and latency to first crossing during the probe test (*n* = 6). (F) Detection of the His6-tagged TAT-PBX1 protein in brain tissue via immunoprecipitation assay. (G) Representative dot blot images showing A11- and 6E10-positive protein levels in mouse brains (*n* = 4). (H) Quantification of A11 and 6E10 levels normalized to those in the WT group (*n* = 4). (I) Images of TUNEL staining in the hippocampus (scale bar: 200 μm, *n* = 3). (J) Quantification of TUNEL-positive regions (*n* = 3). (K) Representative western blot bands for PBX1, Caspase 3, cleaved caspase 3, Bcl-2, and Bax in brain tissues from each group (*n* = 4). (L) Quantitative analysis of protein levels (*n* = 4). Data are expressed as the mean ± SD. Group differences were analyzed using one-way analysis of variance (ANOVA), followed by Holm–Sidak multiple-comparisons tests. *Post hoc* comparisons were performed only after a significant omnibus ANOVA. Behavioral outcomes during the hidden-platform training phase were analyzed using two-way repeated-measures ANOVA, with training day as the within-subject factor and group as the between-subject factor. *P < 0.05, **P < 0.01, and ***P < 0.001. APP/PS1: Amyloid precursor protein/presenilin 1; IP: immunoprecipitation; PBS: phosphate buffered saline; TUNEL: terminal deoxynucleotidyl transferase dUTP nick-end labeling; WT: wild-type.

We then evaluated the therapeutic effect of TAT-PBX1 on memory in APP/PS1 mice. No unintended mortality occurred during the study, and all animals survived as required by the experimental protocol. Compared with WT mice, APP/PS1 mice spent more time and traveled greater distances in the central zone of the OFT, which indicated altered exploratory behavior and an anxiety-like phenotype (*P* < 0.001; **Figure 6A** and **Additional Figure 5A** and **B**). Middle (TP mid) and high doses (TP hi) of TAT-PBX1 significantly reversed these abnormalities, whereas low-dose treatment (TP low) had no effect. In the Y-maze test, APP/PS1 mice spent significantly less time and traveled a significantly shorter distance in the novel arm than WT mice, and their spontaneous alternation percentage and recognition index decreased (*P* < 0.05; **Figure 6B** and **Additional Figure 5C** and **D**). These deficits were significantly alleviated by middle and high doses of TAT-PBX1, whereas the low dose had no effect. In the visible platform phase of the MWM test, no differences in escape latency were observed among groups, which ruled out the presence of visual or motor impairments (*P* > 0.05; **Figure 6D**). During the hidden platform trials (days 2–7), APP/PS1 mice showed a longer escape latency than WT mice (*P* < 0.05; **Figure 6D**). Compared with the APP/PS1 group, all three TAT-PBX1 treatment groups showed a significantly shorter escape latency from day 5 (*P* < 0.05; **Figure 6D**). In the probe trial, APP/PS1 mice displayed a longer escape latency and fewer platform crossings than WT mice (*P* < 0.01; **Figure 6E**), and these changes were effectively reversed by all doses of TAT-PBX1 (*P* < 0.001; **Figure 6C–E**). Collectively, these findings indicate that TAT-PBX1 improves cognitive performance in a dose-dependent manner, with the medium and high doses showing optimal efficacy.

Following the behavioral assessments, the mice were sacrificed for histological evaluations of the brain, liver, and kidneys to assess neuroprotective effects and potential toxicity. No significant differences in body weight were observed among the groups during treatment (*P* > 0.05; **Additional Figure 6A**). No morphological differences in body size (**Additional Figure 6B**), brain (*P* > 0.05; **Additional Figure 6C** and **D**), liver (*P* > 0.05; **Additional Figure 6C** and **E**), or kidneys (*P* > 0.05; **Additional Figure 6C** and **F**) were observed among the groups. Hematoxylin and eosin staining (**Additional Figure 6G**) showed normal hepatic lobular structure across all groups, with radially arranged hepatic cords, polygonal hepatocytes with clear cytoplasm and centrally located nuclei, and intact sinusoids. No vacuolar degeneration, necrosis, inflammatory infiltration, bile duct proliferation, fibrosis, or congestion was observed. Similarly, kidney tissues showed normal glomerular morphology, intact basement membranes, orderly tubular arrangement, and preserved cuboidal tubular epithelial cells without edema or inflammation. These findings indicated that i.p. administration of TAT-PBX1 (up to 2.0 mg/kg) did not induce hepatic or renal toxicity, demonstrating a high level of systemic safety and biocompatibility.

We then examined whether TAT-PBX1 penetrates the brain and elevates PBX1 expression. Immunoprecipitation and Western blot analyses confirmed the presence of His6-tagged PBX1 protein complexes in the brains of TAT-PBX1-treated mice (**Figure 6F**). Furthermore, in mice that received i.p. TAT-PBX1 administration, extensive colocalization of neuronal nuclei and His6 was observed in brain tissue (**Additional Figure 7A**), and His6-positive expression was significantly higher than in untreated mice (**Additional Figure 7B**). In contrast, His6-positive neurons were nearly absent or very sparse in the untreated group. These results demonstrate that TAT-PBX1 effectively reached the target site and was functionally active.

To clarify the therapeutic potential of TAT-PBX1, we investigated its impact on neuropathological alterations and apoptosis-associated proteins in APP/PS1 mice. All three doses of TAT-PBX1 treatment significantly lowered AβO levels in the brains of APP/PS1 mice (*P* < 0.01; **Figure 6G** and **H**). Histologically, neurons in the CA1, CA2, CA3, and dentate gyrus (DG) regions of the WT and PBS groups were densely organized, with intact pyramidal layers and uniformly stained nuclei (**Additional Figure 8**). APP/PS1 mice displayed characteristic AD-like alterations, such as neuronal loss, disorganized pyramidal cell layers, pyknotic nuclei, and diminished neuronal density in the DG. TAT-PBX1 treatment significantly ameliorated these pathological changes in a dose-dependent fashion. The groups that received low and medium doses exhibited better-aligned neurons, fewer pyknotic neurons, and clearer hippocampal layering. The medium-dose group (1.5 mg/kg) displayed the most significant improvement, demonstrating nearly normal hippocampal architecture, whereas the high-dose group displayed no additional enhancement. Terminal deoxynucleotidyl transferase deoxyuridine triphosphate nick end labeling staining demonstrated significantly higher neuronal apoptosis in APP/PS1 mice than in WT mice, which was effectively mitigated by TAT-PBX1 (*P* < 0.05, **Figure 6I** and **J**). Western blot analysis demonstrated a dose-dependent augmentation of PBX1 expression in the hippocampus following TAT-PBX1 treatment (**Figure 6K** and **L**). Furthermore, compared with the APP/PS1 group, the APP/PS1 + TP mid group demonstrated markedly lower cleaved caspase-3/caspase-3 and B-cell lymphoma 2 (Bcl-2)/Bcl-2-associated X protein ratios (**Figure 6K** and **L**). In summary, TAT-PBX1 crosses the blood–brain barrier, protects neurons without toxicity, lowers neuronal apoptosis, and improves cognitive function in APP/PS1 mice. Moreover, a medium dosage showed the optimal effect.

### TAT-PBX1 restores insulin sensitivity in the brain and modulates energy metabolism in APP/PS1 transgenic mice

**Figure 7:**
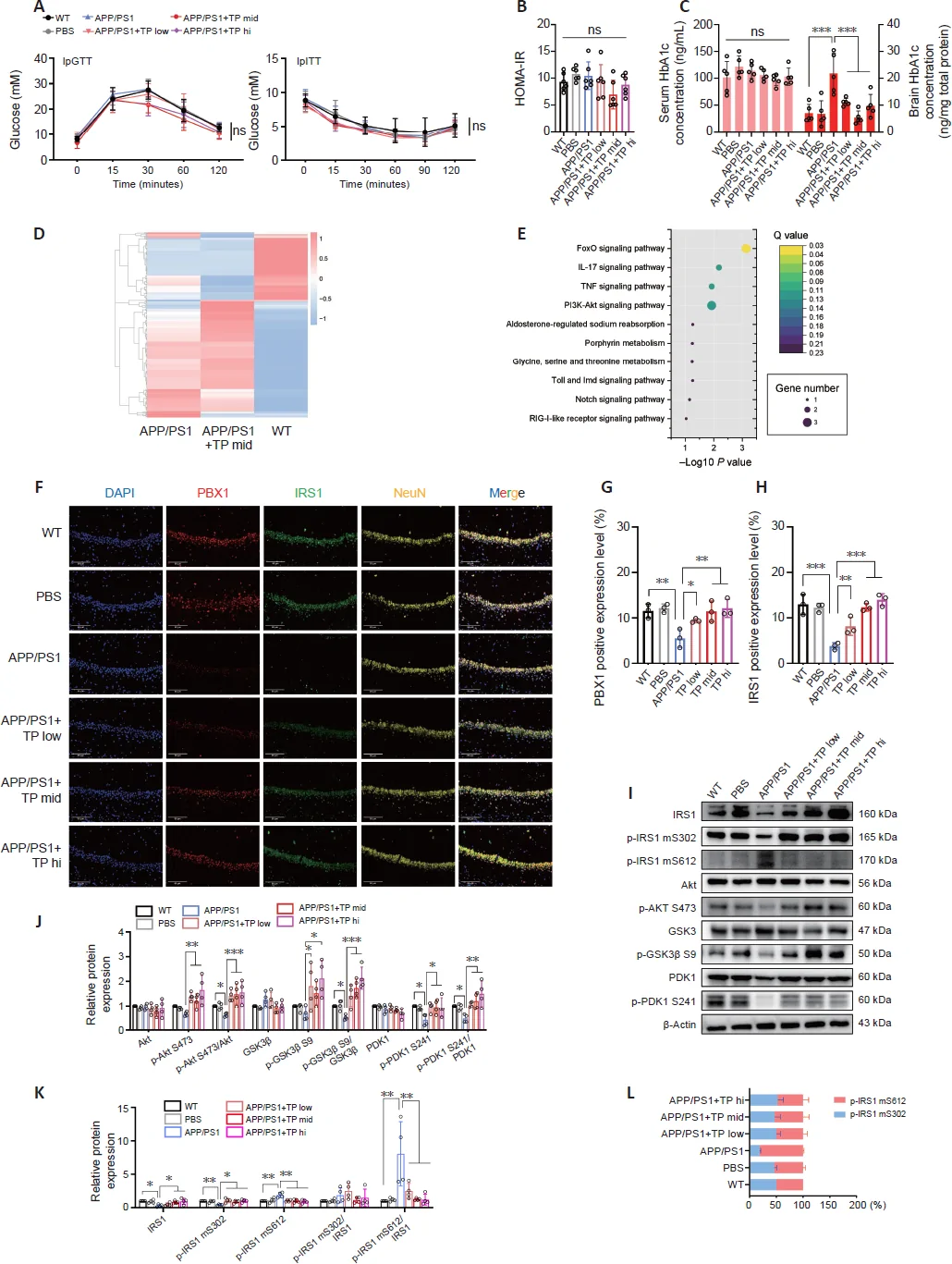
TAT-PBX1 restores insulin resistance in the brains of APP/PS1 mice. (A) Curves for the IpGTT and IpITT (*n* = 6). (B) HOMA-IR index (*n* = 6). (C) HbA1c concentrations in the serum and brain measured by ELISA (*n* = 5). (D) Heatmap of DEGs in brain tissues from WT mice, APP/PS1 mice, and APP/PS1 mice treated with medium-dose TAT-PBX1 (*n* = 4). (E) Bubble plot of the top 10 enriched KEGG pathways in brain tissues from the WT, APP/PS1, and medium-dose TAT-PBX1-treated groups. (F) Representative images of immunofluorescence staining for IRS1 (green), PBX1 (red), and NeuN (yellow) in the hippocampal region of each group (*n* = 3). (G) Quantification of PBX1-positive expression (*n* = 3). (H) Quantification of IRS1-positive expression (*n* = 3). (I) Representative western blot bands for p-IRS1 mS612, p-IRS1 mS302, IRS1, p-Akt S473, Akt, p-GSK3β S9, GSK3β, p-PDK1 S241, and PDK1 (*n* = 4). (J) Quantification of p-Akt S473/Akt, p-GSK3β S9/GSK3β, and p-PDK1 S241/PDK1 ratios (*n* = 4). (K) Quantification of IRS1, p-IRS1 mS612, and p-IRS1 mS302 levels (*n* = 4). (L) Quantification of p-IRS1 mS612/IRS1 and p-IRS1 mS302/IRS1 ratios. Data are expressed as the mean ± SD. Group differences were analyzed using one-way analysis of variance (ANOVA), followed by the Holm–Sidak multiple-comparisons test. *Post hoc* comparisons were performed only after a significant omnibus ANOVA. Blood glucose levels measured at different time points were analyzed using two-way repeated-measures ANOVA. *P < 0.05, **P < 0.01, ***P < 0.001. APP/PS1: Amyloid precursor protein/presenilin 1; ELISA: enzyme-linked immunosorbent assay; GSK3β: glycogen synthase kinase 3 beta; HOMA-IR: homeostatic model assessment for insulin resistance; IpGTT: intraperitoneal glucose tolerance test; IpITT: intraperitoneal insulin tolerance test; IRS1: insulin receptor substrate 1; KEGG: Kyoto Encyclopedia of Genes and Genomes; n.s.: not significant; NeuN: neuronal nuclei; PDK1: 3-phosphoinositide-dependent protein kinase-1; TAT-PBX1: trans-activator of transcription peptide-pre-B-cell leukemia homeobox 1.

To ascertain whether TAT-PBX1 enhances memory by mitigating neuronal insulin resistance in APP/PS1 mice, we initially assessed systemic insulin sensitivity to exclude the effect of confounding variables. I.p. glucose tolerance tests validated normal glucose tolerance in all groups and aligned with Homeostatic Model Assessment of Insulin Resistance values (*P* > 0.05; **Figure 7A** and **B** and **Additional Figure 9A** and **C**). Similarly, i.p. insulin tolerance tests revealed no significant differences in systemic insulin sensitivity among groups (*P* > 0.05; **Figure 7A** and **Additional Figure 9B**), and no notable differences were detected in serum HbA1c levels (*P* > 0.05, **Figure 7C**), which indicated a lack of systemic insulin resistance in these mice. We further evaluated whether TAT-PBX1 diminishes cerebral insulin resistance in APP/PS1 mice. HbA1c levels in the brain tissue of APP/PS1 mice were significantly higher than those in other groups, which indicated that glucose metabolism in the brain of these mice was abnormal (*P* < 0.001; **Figure 7C**). RNA sequencing of the brains of WT, APP/PS1, and medium-dose TAT-PBX1-treated APP/PS1 mice identified 40 differentially expressed genes in the TAT-PBX1 *vs*. APP/PS1 comparisons (fold change > 1.5, *P* < 0.05), which comprised 14 upregulated genes and 26 downregulated genes (**Figure 7D** and **Additional Figures 10A–E**). The Kyoto Encyclopedia of Genes and Genomes enrichment analysis showed that the forkhead box O and phosphatidylinositol 3′-kinase–Akt signaling pathways are crucial and that TAT-PBX1 helps control insulin signaling and energy metabolism in the brain (**Figure 7E**).

We further investigated whether TAT-PBX1 restores brain insulin sensitivity in APP/PS1 mice by promoting IRS1 expression and reducing its pathological phosphorylation. Immunofluorescence staining showed fewer PBX1- and IRS1-positive areas in the hippocampi of APP/PS1 mice than in WT control mice. Notably, treatment with all three dosages of TAT-PBX1 significantly increased IRS1 immunoreactivity (*P* < 0.05; **Figure 7F–H**). Western blot analysis further revealed that the p-Akt/Akt, p-GSK3β/GSK3β, and p-PDK1/PDK1 ratios were significantly lower, the p-IRS1 mS612/IRS1 ratio was significantly higher, and the p-IRS1 mS302/IRS1 ratio was significantly lower in APP/PS1 mice than in WT mice (*P* < 0.05; **Figure 7I–L**). The TAT-PBX1 treatment partially reversed these changes, which indicated reduced severity of insulin resistance in the brain. These results suggested that TAT-PBX1 restores brain insulin sensitivity in APP/PS1 mice by promoting IRS1 expression and reducing its pathological phosphorylation.

**Figure 8:**
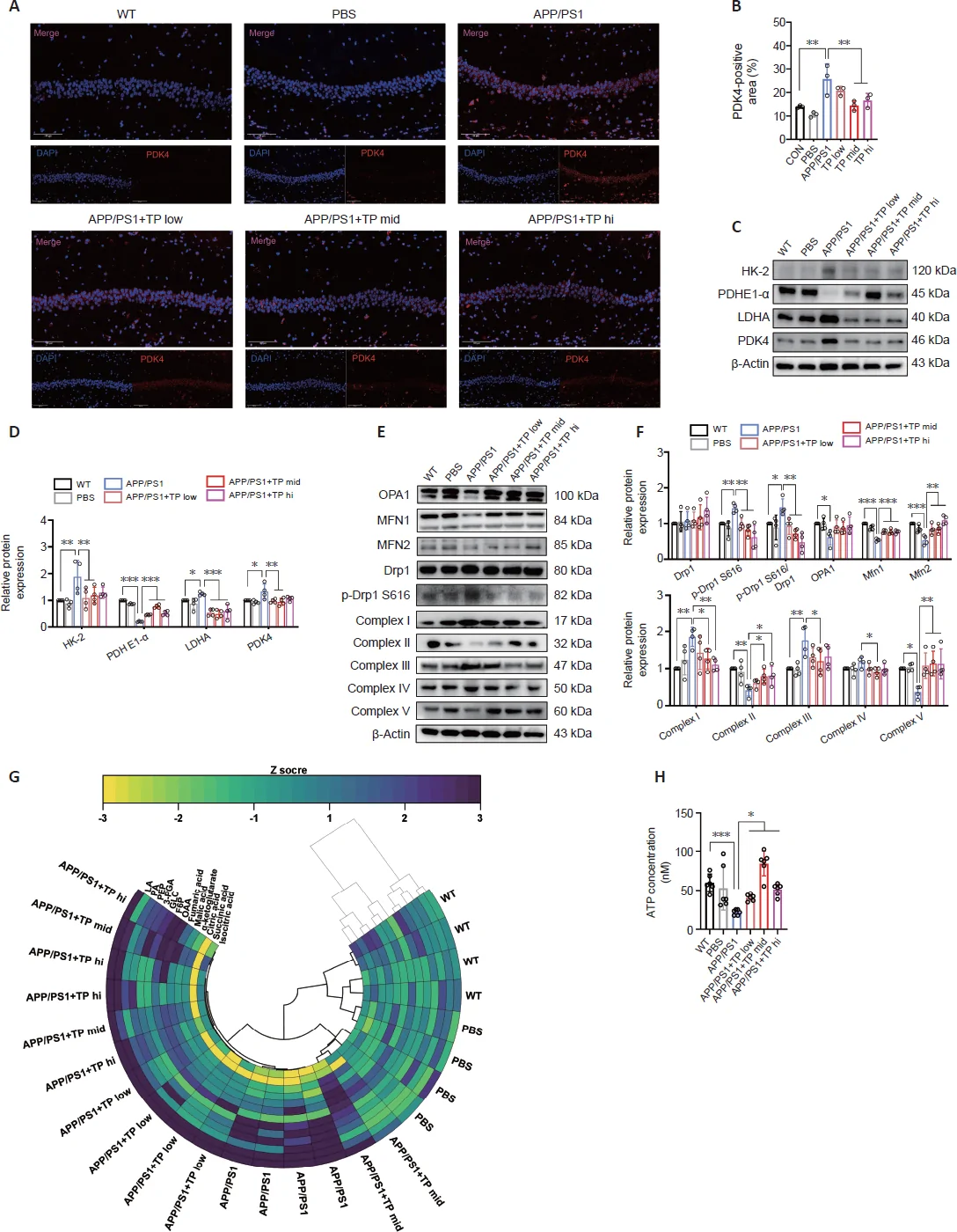
TAT-PBX1 regulates energy metabolism in the brains of APP/PS1 mice. (A) Representative images of immunofluorescence staining showing PDK4-positive regions (in red) in the hippocampus of each group (*n* = 3). (B) Quantification of the PDK4-positive area (*n* = 3). (C) Representative western blot bands for HK-2, PDH E1-α, LDHA, and PDK4 (*n* = 4). (D) Quantification of HK-2, PDH E1-α, LDHA, and PDK4 expression (*n* = 4). (E) Representative western blot bands for OPA1, Mfn1, Mfn2, Drp1, p-Drp1 S616, and mitochondrial respiratory chain complexes I–V (*n* = 4). (F) Quantification of OPA1, Mfn1, Mfn2, Drp1, p-Drp1 S616, and complex I–V levels (*n* = 4). (G) Heatmap of the hierarchical clustering analysis of brain metabolic profiles. (H) Brain ATP concentrations in the different groups (*n* = 6). Data are expressed as the mean ± SD. Group differences were analyzed using one-way analysis of variance (ANOVA), followed by the Holm–Sidak multiple-comparisons test. *Post hoc* comparisons were performed only after a significant omnibus ANOVA. *P < 0.05, **P < 0.01, and ***P < 0.001. APP/PS1: Amyloid precursor protein/presenilin 1; ATP: adenosine triphosphate; Drp1: dynamin-related protein 1; HK-2: hexokinase 2; LDHA: lactate dehydrogenase A; Mfn1: mitofusin 1; Mfn2: mitofusin 2; OPA1: optic atrophy 1; PDH: pyruvate dehydrogenase; PDK4: pyruvate dehydrogenase kinase 4; TAT-PBX1: trans-activator of transcription peptide-pre-B-cell leukemia homeobox 1.

PDK4 is a key mechanism for enhancing mitochondrial OXPHOS and reestablishing metabolic homeostasis. Compared with WT mice, APP/PS1 mice exhibited significantly more PDK4-positive regions in the hippocampus (*P* < 0.01; **Figure 8A** and **B**). The middle and high doses of TAT-PBX1 significantly reduced PDK4 expression (*P* < 0.01; **Figure 8A** and **B**). Western blotting showed that medium-dose TAT-PBX1 treatment decreased the expression of HK2, PDK4, and LDHA but increased the expression of PDH E1-α (*P* < 0.01; **Figure 8C** and **D**). Mitochondrial dynamics are crucial for efficient OXPHOS because they preserve mitochondrial quality and functional integrity. Thus, we further analyzed the levels of proteins associated with mitochondrial dynamics. APP/PS1 mice exhibited higher p-Drp1 S616/Drp1 ratios and lower expression of OPA1 and Mfn1/2 than WT mice, which indicated disrupted mitochondrial dynamics (*P* < 0.05; **Figure 8E** and **F**). TAT-PBX1 treatment at all dosages significantly decreased the p-Drp1 S616/Drp1 ratio and increased Mfn1/2 expression, although OPA1 expression remained unchanged. These results collectively indicate that TAT-PBX1 promotes neuronal OXPHOS by downregulating PDK4; moreover, by regulating mitochondrial dynamics, TAT-PBX1 synergistically enhances mitochondrial OXPHOS function and energy metabolism efficiency in the brains of APP/PS1 mice.

We then conducted gas chromatography-mass spectrometry analysis to quantify the levels of metabolites in the glycolytic and TCA pathways within the hippocampus (**Additional Table 3**). Cluster heatmap analysis showed distinct metabolomic profiles for the WT, PBS, and APP/PS1 groups, with minimal overlap (**Figure 8G**). The metabolic profiles of the three TAT-PBX1 treatment groups were similar. Compared with control mice, APP/PS1 mice displayed higher hippocampal glucose levels and significantly greater concentrations of glycolytic intermediates, such as fructose-6-phosphate, 3-phosphoglycerate, phosphoenolpyruvate, lactate, and pyruvate. Conversely, the levels of TCA cycle intermediates (i.e., citrate, isocitrate, α-ketoglutarate, and succinate) were significantly reduced. TAT-PBX1 treatment reduced the levels of glucose, fructose-6-phosphate, and 3-phosphoglycerate across all treatment groups. Notably, only the medium-dose TAT-PBX1 group (i.e., APP/PS1 + TP mid group) exhibited significant reductions in phosphoenolpyruvate, lactate, and pyruvate levels. Additionally, oxaloacetate levels increased in all TAT-PBX1 treatment groups, whereas citrate, isocitrate, α-ketoglutarate, and malate levels were restored in only the APP/PS1 + TP mid group. Importantly, hippocampal ATP levels were significantly increased in all the TAT-PBX1-treated groups (*P* < 0.05, **Figure 8H**). In summary, these findings indicate that in the brains of APP/PS1 mice, TAT-PBX1 treatment effectively ameliorates insulin resistance, suppresses excessive glycolysis, and enhances OXPHOS. Therefore, restoring energy homeostasis, sustaining ATP supply, and reducing neuronal apoptosis can induce a therapeutic effect on AD.

**Additional Table 3:** GC-MS profiling of glycolytic and oxidative phosphorylation metabolites in mouse brain

Metabolite	WT	PBS	APP/PS1	APP/PS1 + TP low	APP/PS1 + TP mid	APP/PS1 + TP hi
Isocitrate	28.10 ± 6.74**	22.22 ± 1.74*	9.39 ± 3.85	16.93 ± 7.02	23.42 ± 8.29*	22.79 ± 4.62*
Citrate	3.02 ± 1.20*	1.97 ± 0.61	1.21 ± 0.37	2.50 ± 0.32	3.95 ± 0.78***	2.06 ± 0.64
α-Ketoglutarate	58.00 ± 11.24**	31.99 ± 3.95	28.52 ± 10.98	42.16 ± 1.71	60.29 ± 17.54**	38.75 ± 3.83
Malate	0.07 ± 0.01	0.13 ± 0.07	0.13 ± 0.03	0.12 ± 0.01	0.27 ± 0.09*	0.13 ± 0.03
Fumarate	1.20 ± 0.40	1.43 ± 0.61	2.38 ± 1.07	1.70 ± 0.35	3.81 ± 2.34	2.69 ± 1.24
Succinate	1.53 ± 0.14***	1.14 ± 0.26***	0.29 ± 0.25	0.21 ± 0.03	0.45 ± 0.20	0.42 ± 0.26
F6P	0.87 ± 0.21*	0.73 ± 0.03**	1.70 ± 0.69	0.57 ± 0.02**	0.92 ± 0.24*	0.82 ± 0.22*
GLC	1.04 ± 0.25^*^	0.86 ± 0.11**	1.80 ± 0.43	0.96 ± 0.14*	1.28 ± 0.42^*^	1.00 ± 0.26*
3-PGA	31.93 ± 9.02**	33.08 ± 18.07^**^	86.82 ± 22.89	43.27 ± 18.89*	39.00 ± 14.66**	52.53 ± 14.28*
PEP	1.72 ± 0.54	1.23 ± 0.61*	2.89 ± 1.12	1.48 ± 0.41*	0.97 ± 0.29**	2.13 ± 0.41
OAA	1.05 ± 0.37	1.40 ± 0.16	0.77 ± 0.06	2.07 ± 0.40**	1.99 ± 0.42*	2.11 ± 0.83**
PA	12.34 ± 3.47**	11.54 ± 9.08**	59.22 ± 21.36	53.88 ± 11.36	20.49 ± 8.66**	30.99 ± 15.05*
LA	1.71 ± 0.07^***^	1.70 ± 0.16***	5.59 ± 0.24	5.08 ± 0.18	3.61 ± 1.06***	4.87 ± 0.14

Homogeneity of variances was confirmed using Levene’s test. Group differences were analyzed using one-way analysis of variance followed by Holm-Sidak’s multiple-comparisons test (*post hoc* comparisons were performed only if the overall analysis of variance was significant). Metabolite concentrations are expressed in μg/mL. Data meeting variance homogeneity assumptions were analyzed by one-way analysis of variance with Holm-Sidak's multiple comparisons test, while non-homoscedastic data were evaluated using one-way analysis of variance with Dunn's multiple comparisons test. Significance levels are indicated as follows: *P < 0.05, **P < 0.01, ***P < 0.001, *vs*. APP/PS1. F6P: Fructose-6-phosphate; GC-MS: gas chromatography-mass spectrometer; GLC: glucose; LA: lactate; OAA: oxaloacetate; PA: pyruvate; PEP: phosphoenolpyruvate; 3-PGA: 3-phosphoglycerate.

## Discussion

Neuronal insulin signaling is essential for glucose uptake and energy metabolism, and its disruption causes energy deprivation and apoptosis (Sun et al., 2025). Existing therapies, such as exogenous insulin or AβO-targeted strategies, have limited efficacy (Huang et al., 2023). Here, we developed a TAT-PBX1 fusion protein that can be internalized and cross the blood–brain barrier, allowing PBX1 levels to increase in the hippocampus of APP/PS1 mice. Furthermore, we showed that TAT-PBX1 treatment restores insulin sensitivity in AβO-treated SH-SY5Y cells and neurons of APP/PS1 mice, thereby suppressing excessive glycolysis and increasing OXPHOS. In the AD brain, reestablishing energy homeostasis reduces neuronal apoptosis and ameliorates neuropathology. Unlike previous strategies that rely primarily on insulin supplementation, AβO clearance, or direct modulation of metabolic enzyme activity, we proposed and validated a novel therapeutic approach for treating AD (Huang et al., 2023). By upregulating PBX1 to enhance IRS1 expression and reorganize the downstream insulin/mitochondrial metabolic network, our approach restores neuronal energy homeostasis and reduces neuronal apoptosis. Therefore, we have provided foundational cellular and animal data to support future clinical studies.

In mammals, IRS1 is the most studied member of the IRS family and is a critical target of insulin receptor tyrosine kinase. It is widely expressed in the central nervous system, particularly in neurons (Bomfim et al., 2012; Wang et al., 2019). In mice, abnormal phosphorylation of IRS1 at residue hS616 in humans (corresponding to mS612 in mice) or its functional loss leads to AD-like pathology and cognitive deficits (Sun et al., 2024). Phosphorylation at IRS1 hS616 promotes sustained internalization of insulin receptors and reduces insulin sensitivity (Hall et al., 2020). In our study, AβOs increased phosphorylation of IRS1 at hS616/mS612 and decreased phosphorylation of the downstream effectors, Akt, GSK3β, and PDK1, which, in turn, suppressed insulin signaling in neurons. These findings are consistent with those of postmortem analyses of human AD brains (Talbot et al., 2012). Intriguingly, TAT-PBX1 not only altered the phosphorylation levels of Akt and GSK3β but also affected their total protein levels. Following PBX1 activation, the IRS1/Akt pathway was enhanced, which may reflect a positive feedback mechanism that upregulates Akt protein expression. In contrast, the reduction in total GSK3β may be associated with restored insulin signaling because Akt-mediated inhibitory phosphorylation at Ser9 can promote ubiquitin–proteasome-dependent degradation of GSK3β (Baranowski et al., 2024). Therefore, the decrease in GSK3β is more likely to reflect an adaptive regulatory response to insulin signaling recovery than nonspecific downregulation. In addition, increasing IRS1 expression via genetic or phytochemical interventions promotes pancreatic β-cell proliferation, restores insulin secretion, and reduces neuronal apoptosis in the brains of diabetic model mice (Zeng et al., 2024; Massaro et al., 2025). This study provides the first demonstration of targeting brain insulin sensitivity in AD by directly upregulating IRS1. PBX1 binds to and activates the IRS1 promoter, and the resulting increase in IRS1 expression amplifies insulin signaling, enhances neuronal glucose uptake, and supplies more substrates for glycolysis and mitochondrial oxidative metabolism.

Phosphorylation of PDH by PDK4 stops PDH from performing its function, inhibits the conversion of pyruvate to acetyl-CoA, and acts as a key metabolic switch between glycolysis and oxidative phosphorylation (Chalifoux et al., 2023). Insulin blocks PDK4 transcription via glucocorticoid response, forkhead box O1, and estrogen-related receptor elements (Connaughton et al., 2010). Consequently, when insulin signaling is aberrant, PDK4 levels rise, which accelerates the switch from OXPHOS to glycolysis and exacerbates neuronal energy failure. We found that TAT-PBX1 promoted PDH to compete more for pyruvate, which indirectly inhibited LDHA from generating lactate and directed metabolic flow toward mitochondrial OXPHOS. This increase in OXPHOS greatly boosts ETC activity because more acetyl-CoA leads to more TCA cycle flux, which provides the ETC with more reducing equivalents and accelerates proton pumping and ATP synthesis (Hong et al., 2019; Ruan et al., 2023). In pathological conditions characterized by diminished ATP synthase activity, neurons enhance glycolysis to mitigate ATP shortages, thereby diverting energy production from OXPHOS (Formentini et al., 2014; Varghese et al., 2025). TAT-PBX1 not only directs metabolic flux toward mitochondrial OXPHOS but also increases the expression of complex V and decreases the expression of complexes I, III, and IV. This maintains ATP synthesis rates while lowering the risk of reactive oxygen species production, which protects mitochondria against oxidative damage. By preserving the integrity and efficiency of the ETC, TAT-PBX1 provides a stable foundation for sustained enhancement of OXPHOS, offering a promising therapeutic strategy for AD.

## Limitations

Several limitations should be acknowledged. Although we established a preliminary association between PBX1 and cognitive function, causality in AD patients requires validation. Future cohort analyses and neuropathological studies of individuals with MCI/AD are needed. Furthermore, a larger sample size is crucial to elucidate the role of PBX1 in AD progression. Although TAT-PBX1 can effectively penetrate brain tissue and exert neuroprotective effects, its immunogenicity, detailed pharmacokinetic profile, and dosing regimen require further optimization and investigation. Additionally, future studies should explore how the PBX1/IRS1 axis modulates GSK3β activity to influence Tau phosphorylation and Aβ metabolism, given its potential for complementing anti-Aβ and anti-Tau therapies and forming an integrated metabolic-signaling strategy to address AD heterogeneity. Finally, sex is an important biological variable, and considering the neuroprotective effects of estrogen and progestogen, TAT-PBX1 may induce sex-dependent therapeutic effects in female APP/PS1 mice. This should be a key focus of future research.

## Conclusions

We demonstrated that TAT-PBX1 restores insulin sensitivity via IRS1 regulation to remodel neuronal energy metabolism in AD. Our findings offer a foundation for PBX1-based preclinical therapies and highlight a promising metabolic energy-targeted approach for treating neurodegenerative disorders.

## Additional files:

***Additional Figure 1:*** *Exploratory evaluation of TAT-PBX1 administration routes.*

Additional Figure 1 Exploratory evaluation of TAT-PBX1 administration routes.
PBX1 concentrations in the plasma and brain tissue of WT mice at 0, 5, 15, 30 minutes, and 1, 2, 4, 8, 10, and 24 hours following the intraperitoneal or intragastric administration of TAT-PBX1 (*n* = 3 /time point). Data are expressed as the mean ± SD. PBX1: Pre-B-cell leukemia homeobox 1; WT: wild type.

***Additional Figure 2:*** *Cytotoxicity of AβO prepared under different conditions.*

Additional Figure 2 Cytotoxicity of AβO prepared under different conditions.
(A) SH-SY5Y cells were co-incubated with the AβO sample (prepared at 4°C for 24 hours) (*n* = 6); (B) SH-SY5Y cells were co-incubated with the AβO sample (prepared at 4 °C for 48 h) (*n* = 6); (C) SH-SY5Y cells were co-incubated with the AβO sample (prepared at 37°C for 24 hours) (*n* = 6); (D) SH-SY5Y cells were co-incubated with the AβO sample (prepared at 37°C for 48 hours) (*n* = 6). (E) Representative Western blot bands for Caspase 3, cleaved Caspase 3, and Cyto C expression (*n* = 4). (F) Quantitative analysis of Caspase 3, cleaved Caspase 3, and Cyto C protein expression (*n* = 4). Cell viability was assessed using the CCK-8 assay and normalized to the blank group. Data are expressed as the mean ± SD. Group differences were analyzed using one-way analysis of variance (ANOVA), followed by Holm–Sidak multiple-comparisons tests. *Post hoc* comparisons were performed only after a significant omnibus ANOVA. Significance levels are indicated as follows: *P < 0.05, **P < 0.01, ***P < 0.001. AβO: Amyloid beta oligomer; Cyto C: Cytochrome c.

***Additional Figure 3:*** *Validation of insulin resistance model in SH-SY5Y cells.*

Additional Figure 3 Validation of insulin resistance model in SH-SY5Y cells.
(A) Representative Western blot bands showing the levels of Akt, Akt phosphorylated at Ser473 (p-Akt S473), GSK3β, and GSK3β phosphorylated at Ser9 (p-GSK3β S9) in SH-SY5Y cells stimulated with varying concentrations of insulin for different durations (*n* = 4). (B) Quantitative analysis of the protein expression levels (*n* = 4). Data are expressed as the mean ± SD. Group differences were analyzed using one-way analysis of variance (ANOVA), followed by Holm–Sidak multiple-comparisons tests. *Post hoc* comparisons were performed only after a significant omnibus ANOVA. Significance levels are indicated as follows: *P < 0.05, **P < 0.01, ***P < 0.001. ANOVA: Analysis of variance; GSK3β: Glycogen synthase kinase 3 beta; Ins: Insulin.

***Additional Figure 4:*** *Effects of TAT-PBX1 on insulin signaling–related proteins.*

Additional Figure 4 Effects of TAT-PBX1 on insulin signaling–related proteins.
Quantitative analysis of IRS1 phosphorylated at Ser616 (p-IRS1 hS616), IRS1 phosphorylated at Ser307 (p-IRS1 hS307), IRS1, p-Akt S473, Akt, p-GSK3β S9, GSK3β, PDK1 phosphorylated at Ser241 (p-PDK1 S241), and PDK1 protein expression after treatments with AβOs and/or TAT-PBX1 (*n* = 4). Data are expressed as the mean ± SD. Group differences were analyzed using one-way analysis of variance (ANOVA), followed by Holm–Sidak multiple-comparisons tests. Post hoc comparisons were performed only after a significant omnibus ANOVA. Significance levels are indicated as follows: *P < 0.05, **P < 0.01, ***P < 0.001. AβO: Amyloid beta oligomer; GSK3β: glycogen synthase kinase 3 beta; IRS1: insulin receptor substrate 1; PDK1: 3-phosphoinositide-dependent protein kinase-1; TP: TAT-PBX1.

***Additional Figure 5:*** *TAT-PBX1 improves working memory and exploratory memory in APP/PS1 mice.*

Additional Figure 5 TAT-PBX1 improves working memory and exploratory memory in APP/PS1 mice.
(A) Representative trajectories and heatmaps from the open field test (*n* = 6). (B) Quantification of distance traveled in the center area, total time spent in the center, and total distance traveled in the OFT (*n* = 6). (C) Representative trajectories and heatmaps from the Y-maze test (*n* = 6). (D) Time spent and distance traveled in the novel arm during the Y-maze test (*n* = 6). Data are expressed as the mean ± SD. Group differences were analyzed using one-way analysis of variance (ANOVA), followed by Holm–Sidak multiple-comparisons tests. *Post hoc* comparisons were performed only after a significant omnibus ANOVA. Significance levels are indicated as follows: *P < 0.05, **P < 0.01, ***P < 0.001. OFT: Open field test.

***Additional Figure 6:*** *Effects of TAT-PBX1 treatment on the brain, liver, and kidney of APP/PS1 mice.*

Additional Figure 6 Effects of TAT-PBX1 treatment on the brain, liver, and kidney of APP/PS1 mice.
(A) Body weight changes during TAT-PBX1 administration (*n* = 6). (B) Representative morphological images of mice from each group. (C) Representative anatomical images of the whole brain, liver, and kidneys from each group. (D) Brain weight and brain-to-body weight ratio (*n* = 6); (E) Liver weight and liver-to-body weight ratio (*n* = 6). (F) Kidney weight and kidney-to-body weight ratio (*n* = 6). (G) H&E staining of liver and kidney sections from each group. Scale bar: 100 μm. Data are expressed as the mean ± SD. Group differences were analyzed using one-way analysis of variance (ANOVA), followed by Holm–Sidak multiple-comparisons tests. *Post hoc* comparisons were performed only after a significant omnibus ANOVA. H&E: Hematoxylin and eosin staining; n.s.: not significant.

***Additional Figure 7:*** *Intraperitoneally injected TAT-PBX1 is capable of entering neuronal cells.*

Additional Figure 7 Intraperitoneally injected TAT-PBX1 is capable of entering neuronal cells.
(A) Representative images of brain sections from the mice in each group showing His tag (in red)- and NeuN (in green)-positive areas; scale bar, 200 μm. (B) Quantification of the proportion of His tag-positive areas (*n* = 4). Data are expressed as the mean ± SD. Group differences were analyzed using one-way analysis of variance (ANOVA), followed by Holm–Sidak multiple-comparisons tests. *Post hoc* comparisons were performed only after a significant omnibus ANOVA. Significance levels are indicated as follows: *P < 0.05, **P < 0.01. NeuN: Neuronal nuclei.

***Additional Figure 8:*** *H&E staining of the hippocampus from each group of mice.*

Additional Figure 8 H&E staining of the hippocampus from each group of mice.
Representative H&E-stained sections of the hippocampus from the different treatment groups. Scale bars: CA1, CA2 and CA3 (50 μm), DG (100 μm). DG: Dentate gyrus; H&E: hematoxylin and eosin staining.

***Additional Figure 9:*** *Effects of TAT-PBX1 on glucose tolerance and insulin resistance in APP/PS1 mice.*

Additional Figure 9 Effects of TAT-PBX1 on glucose tolerance and insulin resistance in APP/PS1 mice.
(A) Area under the curve (AUC) for the IpGTT (*n* = 6). (B) AUC for the IpITT (n = 6). (C) Fasting serum insulin concentrations (*n* = 6). Data are expressed as the mean ± SD. Group differences were analyzed using one-way analysis of variance (ANOVA), followed by Holm–Sidak multiple-comparisons tests. *Post hoc* comparisons were performed only after a significant omnibus ANOVA. AUC: Area under the curve; IpGTT: Intraperitoneal glucose tolerance test; IpITT: Intraperitoneal insulin tolerance test; n.s.: not significant.

***Additional Figure 10:*** *Analysis of DEGs.*

Additional Figure 10 Analysis of DEGs.
(A) Violin plots showing the gene expression distribution across the experimental groups. (B) Bar graph summarizing the number of DEGs identified in each comparison group. (C–E) Volcano plots depicting the DEGs. DEG: Differentially expressed gene.

***Additional Table 1:*** *Pharmacokinetic parameters of TAT-Pre-B-cell leukemia transcription factor 1 (PBX1) in mice following intraperitoneal (i.p.) injection and oral gavage administration.*

***Additional Table 2:*** *Primary antibodies used in this study.*

***Additional Table 3:*** *GC-MS profiling of glycolytic and oxidative phosphorylation metabolites in mouse brain.*

***Additional file 1:*** *Methodological details.*

Additional file 1 Methodological details

## Data Availability

*All relevant data are within the paper and its Additional files. Detailed datasets regarding RNA sequencing are available from the corresponding author upon reasonable request.*
