# Supplementary material for: Trans-activator of transcription-pre-B-cell leukemia transcription factor 1 alleviates Alzheimer’s disease by reducing neuronal insulin resistance and restoring energy homeostasis
Source: Neural Regen Res. 2026 Feb 5;21(9):4390–405. doi: 10.4103/NRR.NRR-D-25-01313 (PMC13557548; doi:10.4103/NRR.NRR-D-25-01313)
Supplement: Supplementary file 1 [file NRR-21-4390_Suppl1.pdf]

Additional file 1 Methodological details

Cell viability assay

Cell viability was assessed using the CCK-8 assay kit (SC119, Sevenbio, Beijing, China). Briefly, cells were seeded in 96-well plates at a density of 8 × 103 cells/well. After treatment, CCK-8 reagent was added directly to the culture medium, and cells were incubated at 37°C for 1.5 hours. Absorbance at 450 nm was measured using a microplate reader.

Flow cytometry analysis

Apoptosis was assessed using the BD Pharmingen™ FITC Annexin V Apoptosis Detection Kit (556547, BD Biosciences, San Jose, CA, USA) according to the manufacturer's instructions. Briefly, cells were washed twice with pre-chilled PBS and resuspended in 1× binding buffer at a concentration of 1×10^6 cells/mL. FITC-conjugated Annexin V and propidium iodide were added to the cell suspension, followed by gentle vortexing. The samples were incubated in the dark at room temperature for 15 minutes and analyzed using a flow cytometer (BD FACSCalibur, BD Biosciences, San Jose, CA, USA).

Measurement of cellular glucose uptake

The glucose uptake capacity of SH-SY5Y cells was evaluated using a commercial glucose assay kit based on the glucose oxidase method (A154-1-1, Nanjing Jiancheng, Jiangsu, China). Briefly, culture supernatants were collected from SH-SY5Y cells treated with insulin, AβO, and TAT-PBX1. After centrifugation to remove cell debris, glucose concentrations in the supernatants were measured according to the manufacturer's protocol. Additionally, 2-NBDG (2-(N-(7-nitrobenz-2-oxa-1,3-diazol-4-yl)amino)-2-deoxyglucose, S8914, Selleck, shanghai, China), a fluorescent glucose analog, was used to assess glucose uptake. 2-NBDG enters cells via glucose transporters but is not metabolized, allowing intracellular accumulation. The intensity of its fluorescence signal is proportional to the glucose uptake capacity of cells and is widely used as an indicator of cellular glucose metabolism. SH-SY5Y cells were seeded into black, clear-bottom 96-well and 6-well plates at a density of 1×10^4 cells/well and cultured at 37°C with 5% CO_2 until they reached approximately 80% confluency. A working solution of 2-NBDG (100 μM) was prepared in serum-free 1:1 mix MEM/F12 medium using the fluorescent probe. The culture medium was discarded and replaced with the 2-NBDG working solution. Cells were incubated at 37°C for 30 minutes, and images were acquired using a Biotek Cytation multi-mode reader (Agilent, Santa Clara, CA, USA). Fluorescence intensity was quantified to evaluate glucose uptake.

ATP quantification

Cellular and brain tissue ATP levels were quantified using a commercial ATP Assay Kit (S0026, Beyotime, Shanghai, China), according to the manufacturer's instructions. For cell samples, cells were harvested by trypsinization and centrifuged at 1000 rpm for 5 minutes. The cell pellet was washed twice with pre-chilled PBS, centrifuged, and the supernatant discarded each time. Subsequently, 100 μL of ATP lysis buffer was added per 10^6 cells. The suspension was vortexed thoroughly and lysed on ice for 10 minutes, followed by centrifugation at 12,000 rpm at 4°C for 5 minutes to collect the supernatant. For tissue samples, approximately 200 μL of ATP lysis buffer was added to 20 mg of mouse brain tissue. The tissue was mechanically disrupted in an ice bath using zirconium beads. After standing at room temperature for 3–5 minutes to eliminate background luminescence, the supernatant was collected for ATP quantification.

Lentiviral and siRNA Transfection

The lentiviral vector encoding human PBX1 (pLV-CMV-CDNA-IRES-EGFP, Youbio, Shanghai, China) was used for stable overexpression. Briefly, 293T cells were seeded in T75 flasks and co-transfected with 10 μg of the lentiviral construct, 7.5 μg of pMD2.G, and 2.5 μg of psPAX2 (Addgene, F Cambry, MA, USA) using Lipofectamine 3000 (L3000008, Invitrogen, Carlsbad, CA, USA). Viral supernatants were collected at 48- and 72-hour post-transfection and filtered through 0.22 μm syringe filters (SLGP033RB, Millipore, Billerica, MA, USA). SH-SY5Y cells were seeded at a density of 5×10^4 cells/well in 24-well plates. When cells reached approximately 50% confluence, they were infected with viral particles in the presence of 8 μg/mL polybrene (28728-55-4, Santa Cruz Biotechnology, Santa Cruz, CA, USA). After 24 hours of incubation at 37°C, the medium was replaced with fresh complete culture medium. For siRNA transfection, SH-SY5Y cells were transfected with a small interfering RNA targeting IRS1 (siIRS1) or a negative control (Vector) using Lipofectamine 2000 (11668027, Invitrogen, Carlsbad, CA, USA). siRNA and Lipofectamine 2000 were mixed at a 1:1 volume ratio in Opti-MEM (31985070, Gibco, Grand Island, NY, USA) and incubated at

room temperature for 20 minutes. Cells were seeded in 24-well plates and allowed to reach 60% confluence, after which the medium was replaced with serum-free medium and the siRNA complexes (final concentration: 50 nM) were added. After 9 hours of incubation, the medium was replaced with complete medium. Cells were harvested 48 hours later for Western blot analysis of IRS1 expression. The siRNA sequences targeting IRS1 were as follows: Sense: 5′-CCUACUACUCAUUGCCAAGAU/dTdT-3′; Antisense: 5′-AUCUUGGCAAUGAGUAGUAGG/dTdT-3′.

Dual-luciferase reporter assay

The promoter region of IRS-1 was synthesized and cloned into the pGL4.19 luciferase reporter vector (Sangon Biotech, Shanghai, China). SH-SY5Y cells were co-transfected with pGL4.19 plasmids containing varying lengths or mutant sequences of the IRS-1 promoter and the pRL-TK plasmid at a ratio of 50:1 using Lipofectamine 2000 (11668027, Invitrogen, Carlsbad, CA, USA). Luciferase activity was measured using the Dual-Luciferase Reporter Assay Kit (CW9312M, Cwbio, Jiangsu, China) on the Biotek Cytation multifunctional imaging system (Agilent, Santa Clara, CA, USA). Firefly luciferase activity was normalized to Renilla luciferase activity for each sample to control for transfection efficiency.

Chromatin immunoprecipitation followed by qPCR

Chromatin immunoprecipitation (ChIP) assays were performed using the Pierce™ Magnetic ChIP Kit (26157, Thermo Scientific, Waltham, MA, USA) according to the manufacturer’s protocol. Briefly, cells were cultured to approximately 80% confluence and crosslinked with 1% formaldehyde at room temperature for 10–15 minutes. Crosslinking was quenched by the addition of glycine to a final concentration of 0.125 M, followed by incubation at room temperature for 5 minutes. Cells were washed twice with pre-chilled PBS containing 1% protease inhibitor, scraped, and centrifuged at 500 × g for 5 minutes at 4°C. The pellet was resuspended in 1 mL of ChIP Lysis Buffer, transferred to microcentrifuge tubes, and vortexed briefly. After complete cell lysis, chromatin was sheared by sonication, followed by centrifugation at 12,000 rpm for 10 minutes at 4°C. The supernatant containing sheared chromatin was collected, and 50 μL was reserved as an input control. The remaining 450 μL of chromatin was incubated with either anti-PBX1 antibody or control IgG overnight at 4°C with rotation. The next day, Protein G magnetic beads were added to each immunoprecipitation tube and incubated for 2 hours at 4°C with rotation to capture immune complexes. Both the immunoprecipitated samples and input controls were incubated with 200 μL of elution buffer (1% SDS, 0.1 M $NaHCO_3$) at 65 °C for 6 hours or overnight to reverse crosslinks. DNA was purified using the Tiangen DNA Purification Kit (DP214, Tiangen, Beijing, China) and subjected to downstream ChIP-qPCR or ChIP-PCR analysis. The primer sequences used for IRS1 promoter detection were as follows: IRS1-Primer-F: 5′-ACCCTTCTACCCCCACCTT; IRS1-Primer-R: 5′-GAGGCCTGCTGATTGGTG.

Electrophoretic mobility shift assay

The promoter region of the IRS1 gene, predicted by JASPAR to contain potential PBX1-binding sites, was amplified by PCR and subsequently labeled at the 3′ end with biotin using the Biotin 3′ End DNA Labeling Kit (GS008, Beyotime, Shanghai, China), following the manufacturer’s instructions. The labeled oligonucleotides were annealed prior to use. The sequences of the sense strands of the probes used were as follows: Wild-type probe: 5′-GCTTGAAAAACAGGCCATCCCACCAATCAGCAGGCCTCCCCGGGAAACTC-3′; Mutant probe: 5′-GCTTGAAAAACAGGCCATCTTGTTGGCTGATAGGCCTCCCCGGGAAACTC-3′. For Electrophoretic Mobility Shift Assay (EMSA), 10 μg of recombinant PBX1 protein (18204-1-AP, Proteintech, Wuhan, China) was incubated with 10 nM biotin-labeled oligonucleotide in binding buffer (GS009, Beyotime, Shanghai, China) for 20 minutes at room temperature to allow specific DNA–protein interactions and reduce nonspecific binding. For competitive EMSA, a 100× molar excess of unlabeled ("cold") wild-type probe was pre-incubated with PBX1 protein for 20 minutes at room temperature before the addition of 10 nM biotin-labeled probe. For supershift assays, 1 μg of anti-PBX1 antibody was added after DNA–protein binding and incubated for an additional 30 minutes at room temperature. Negative controls included only the wild-type biotin-labeled probe without protein or antibody. Samples were resolved on a 6% native polyacrylamide gel that had been pre-electrophoresed at 80 V for 10 minutes. Electrophoresis was conducted on ice to maintain complex stability. Following separation, DNA–protein complexes were transferred onto a nylon membrane (Pall, 60336, NY, USA) and UV crosslinked at 120 mJ/cm² to immobilize DNA. The membrane was blocked in 5% non-fat milk for 30 minutes, incubated with streptavidin-HRP (1:1000 dilution) at room temperature for 1 hour, and developed using enhanced chemiluminescence (ECL) reagents (180-5001, Tanon, Shanghai, China).

Pharmacokinetic study

The whole blood was collected into 1.5 mL EDTA-containing anticoagulant tubes and centrifuged at 4,000 × g for 5 minutes at 4°C. Mouse brain tissues were harvested on ice and immediately stored at −80 °C. Plasma and brain tissue concentrations of PBX1 were measured using Enzyme-Linked Immunosorbent Assay (ELISA) at the following time points: 0, 5, 15, 30 minutes, 1, 2, 4, 8, 24, and 36 hours (n = 3 mice per time point). The time to reach maximum plasma concentration (T_{max}) and the maximum concentration (C_{max}) were used to evaluate the rate of drug absorption into systemic circulation. The area under the plasma concentration-time curve (AUC) was used to reflect the extent of drug absorption. The terminal elimination rate constant (λ_z) was calculated using log-linear regression of the terminal phase of the concentration-time curve, and the elimination half-life ($T_{½}$) was determined using the formula: $T_{½} = \ln(2)/\lambda_z$. C_{last} refers to the last quantifiable drug concentration. $AUC_{(0–t)}$, the area under the curve from time zero to the last measurable concentration, was calculated directly from the concentration-time data. The area under the concentration-time curve extrapolated to infinity, $AUC_{(0–\infty)}$, was calculated using the following equation: $AUC_{(0–\infty)} = AUC_{(0–t)} + C_{last}/\lambda_z$. The mean residence time (MRT) represents the average time a drug molecule remains in the body, encompassing the entire process from administration to complete elimination. It is calculated as the ratio of the Area Under the Moment Curve (AUMC) to AUC. Where AUMC is defined as: AUMC= $\int_0^\infty t \times C(t)dt$ with *C(t)* representing drug concentration at time *t*, and $t \times C(t)$ indicating the product of time and concentration. The clearance (CL) represents the volume of plasma from which the drug is completely removed per unit time, calculated as: CL = Div/$AUC_{(0–\infty)}$, where *Div* is the administered dose. The apparent volume of distribution (V_d) reflects the proportionality between the total amount of drug in the body and its plasma concentration at steady state, and is calculated as: V_d = Div/($AUC_{(0–\infty)} \times \lambda_z$).

Dot blot

Nitrocellulose membranes (66485, Pall, NY, USA) were cut into 1 cm-wide strips. A 2 μL aliquot of each sample was carefully dotted onto the membrane with 0.5 cm spacing between spots. Freshly dissolved HFIP-treated $A\beta_{1–42}$ monomers (800 ng) were spotted as the positive control, and an equal volume of PBS was used as the negative control. Membranes were air-dried at room temperature for 5 minutes to ensure complete absorption. The dried membranes were blocked by immersion in 5% BSA for 30 minutes at room temperature, followed by three washes in TBST (Tris-buffered saline with 0.1% Tween-20) on a rocker, 10 minutes per wash. The membranes were then incubated for 2 hours at room temperature with primary antibodies against Aβ oligomers (A11, AHB0052, Invitrogen, Carlsbad, CA, USA) and total Aβ (6E10, MA5-48043, Invitrogen, Carlsbad, CA, USA). Following primary antibody incubation, membranes were washed three times with TBST (10 minutes per wash) and then incubated with HRP-conjugated secondary antibodies at room temperature for 1 hour. After secondary incubation, the membranes were again washed three times with TBST, 10 minutes each. ECL substrate was applied evenly across the membrane surface, and signals were visualized using a chemiluminescence imaging system. Band intensities were quantified by densitometric analysis using ImageJ software to obtain semi-quantitative results.

Hematoxylin and eosin staining

Following euthanasia, the mouse brain, liver, and kidney tissues were rapidly harvested and rinsed with physiological saline to remove blood. Tissues were immediately fixed in 4% paraformaldehyde at room temperature for 48 hours. After fixation, tissues were dehydrated through a graded ethanol series, cleared with xylene until fully transparent, and embedded in paraffin. Sections were dewaxed and rehydrated sequentially using xylene, absolute ethanol, and 70% ethanol. The slides were then stained with hematoxylin solution for 5–10 minutes, rinsed with running tap water, and differentiated in 1% acid ethanol. After bluing under running water for 10 minutes, sections were counterstained with eosin for 2 minutes, rinsed again, dehydrated through a graded ethanol series, and cleared with xylene. Slides were mounted with neutral resin and coverslipped. Histological images were observed and captured using a light microscope (CX33, Olympus, Tokyo, Japan).

TUNEL staining

Neuronal apoptosis in the mouse hippocampus was assessed using a TUNEL assay kit (E-CK-A324, Elabscience, Wuhan, China). Briefly, whole mouse brains were harvested and fixed in 4% paraformaldehyde for 48 hours, followed by paraffin embedding and coronal sectioning into 3–4 μm slices. Paraffin sections were dewaxed twice with xylene, rehydrated through a graded ethanol series (100%, 95%, 90%, 80%, and 70%), and rinsed twice with PBS. Tissue sections were then treated with Proteinase K working solution at 37°C for 15–30 minutes, followed by two PBS washes. The TUNEL reaction mixture was prepared by mixing 50 μL of TdT enzyme with 450 μL of fluorescein-labeled dUTP solution. Sections were incubated with the reaction mixture at 37°C for 60 minutes in a humidified, light-protected chamber. After incubation, sections were washed with PBS and counterstained with DAPI at room

temperature for 10–20 minutes in the dark. Following DAPI staining, sections were mounted with an anti-fade mounting medium or neutral resin and covered with a coverslip. Images were acquired under a fluorescence microscope.

Immunofluorescence staining

For cell-based immunofluorescence

SH-SY5Y cells were cultured to 60–80% confluence and then treated with either TAT-PBX1 (30 µg/mL) or recombinant PBX1 protein (30 µg/mL) for 12 hours. After treatment, the culture medium was removed, and cells were washed three times with pre-chilled PBS. Cells were fixed with 4% paraformaldehyde at room temperature for 20 minutes, followed by three washes with PBS. Permeabilization was performed using 0.1% Triton X-100 for 10 minutes at room temperature, followed by three additional PBS washes. Cells were blocked with 1% bovine serum albumin (BSA) for 1 hour at room temperature and then incubated overnight at 4°C in a humidified chamber with a primary antibody mixture containing anti-HA and anti-His antibodies. The next day, cells were washed three times with PBS and incubated with the appropriate fluorophore-conjugated secondary antibodies for 1.5 hours at room temperature in the dark. After three PBS washes in the dark, cells were mounted with DAPI-containing antifade mounting medium (P0131, Beyotime, Shanghai, China) and imaged using a fluorescence microscope.

For tissue-based immunofluorescence

Mouse brain tissues were paraffin-embedded, dewaxed, and rehydrated as previously described. Antigen retrieval was performed by immersing sections in preheated sodium citrate buffer (pH 6.0), bringing to a boil, and maintaining at 95°C for 15 minutes using a microwave oven, followed by natural cooling to room temperature. Sections were then permeabilized with 0.1% Triton X-100 for 15 minutes and washed three times with PBS. After blocking with 1% BSA at 37°C for 1 hour in a humidified chamber, tissue sections were incubated overnight at 4°C with primary antibodies against PBX1, IRS1, and NeuN. On the following day, sections were washed three times with PBS and incubated with fluorophore-conjugated secondary antibodies at room temperature for 1 hour in the dark. After three final PBS washes, sections were mounted with DAPI-containing antifade mounting medium (P0131, Beyotime, Shanghai, China) and imaged under a fluorescence microscope (Olympus, Tokyo, Japan). At least three distinct areas were imaged per section. Fluorescence intensity was quantified using ImageJ software.

ELISA

Brain tissues were homogenized in pre-chilled PBS and centrifuged at 12,000 × g for 15 minutes at 4°C. Protein concentration in the supernatant was determined using a BCA protein assay kit (ZJ101, Epizyme, Shanghai, China). For blood samples, whole blood was centrifuged at 3000 rpm for 15 minutes using microcentrifuge tubes with or without anticoagulant to obtain plasma and serum, respectively. The levels of PBX1 in human and mouse plasma and brain tissues were measured using ELISA kits (human: ABIN1149118; mouse: ABIN6212126; antibodies-online, Aachen, Germany), according to the manufacturers' protocols. Serum insulin (JLC3967, Gelatins, Jiangxi, China) and HbA1c levels (JLC3751, Gelatins, Jiangxi, China) in mice were also determined by ELISA following the corresponding kit instructions.

RNA extraction and sequencing

Total RNA was extracted from mouse brain tissue samples (n=4 per group) using TRIzol reagent (15596018CN, Invitrogen Carlsbad, CA, USA). Following DNase I treatment, RNA integrity was assessed using an Agilent 2100 Bioanalyzer (RIN >7.0). Stranded mRNA libraries were prepared with the TruSeq Stranded mRNA Library Prep Kit (Illumina), with fragment sizes selected at 300-500 bp. Library quality was validated by Qubit 3.0 Fluorometer (Thermo Fisher Waltham, MA, USA) and qPCR quantification. Paired-end sequencing (2×150 bp) was performed on the Illumina NovaSeq 6000 platform. Raw sequencing data were deposited in the NCBI SRA database (accession: PRJNA1289025). Low-quality reads were filtered using fastp, and raw data quality was evaluated with FastQC v0.11.2. To detect potential contamination, 10,000 randomly selected clean reads were aligned using Kraken2, followed by taxonomic abundance estimation with Bracken. Clean reads were then aligned to the mouse reference genome (GRCm39) using HISAT2 v2.2.1. Transcript assembly and quantification were performed with StringTie v2.2.1, and differential expression analysis was conducted using DESeq2 (cutoff: $|\log2FC| > 1.5$, $P < 0.05$). Functional annotation of genes was performed based on GO and KEGG databases (clusterProfiler v4.0).

Gas chromatography–mass spectrometry

Metabolites related to glycolysis and the TCA cycle in mouse brain tissues were analyzed by Gas chromatography–mass

spectrometry (GC-MS). Briefly, frozen brain tissues stored at −80°C were weighed after grinding in liquid nitrogen. Tissue powders were extracted in an ethyl acetate:chloroform (2.5:1, v/v) mixture at a 1:10 (w/v) ratio and stored at −80°C overnight. Samples were then centrifuged at 12,000 × g for 10 minutes at 4°C, and 150 μL of the supernatant was collected and subjected to vacuum freeze-drying (cold trap temperature: −95°C; shelf temperature: −20°C; vacuum pressure: 1 Pa). After lyophilization, samples were derivatized. First, methoxyamine hydrochloride (89803, Sigma, USA) dissolved in pyridine (270407, Sigma, USA) was added for methoximation at 37°C for 1 h using a nitrogen blow concentrator. Subsequently, N,O-bis(trimethylsilyl)trifluoroacetamide (BSTFA) with 1% TMCS (15222, Sigma, St. Louis, MO, USA) was added and incubated at 60 °C for 1 h for silylation. GC-MS analysis was performed using an Agilent 8890A gas chromatograph coupled to a 7000D mass spectrometer. The separation was achieved on a DB-5MS capillary column (Agilent, Santa Clara, CA, USA). High-purity nitrogen (99.999%) was used as the carrier gas at a flow rate of 1 mL/min. The inlet temperature was set to 260 °C, and the quadrupole temperature to 150°C. Mass spectra were acquired in the range of 50–550 m/z. The GC oven temperature was programmed as follows: held at 80°C for 2 minutes, ramped at 10 °C/min to 180°C, then at 5 °C/min to 240°C, and finally at 25 °C/min to 290°C. Raw mass spectral data were denoised and deconvoluted using AMDIS software. Metabolites were identified by comparing spectra with entries in the Fiehn and NIST compound libraries and quantified by relative peak area.
